# Single molecule magnets of cobalt and zinc homo- and heterometallic coordination polymers prepared by a one-step synthetic procedure†

**DOI:** 10.1039/d0ra09132d

**Published:** 2020-12-21

**Authors:** Núria Portolés-Gil, Silvia Gómez-Coca, Oriol Vallcorba, Gregorio Marbán, Núria Aliaga-Alcalde, Ana López-Periago, José A. Ayllón, Concepción Domingo

**Affiliations:** Instituto de Ciencia de Materiales de Barcelona (CSIC) Campus UAB 08193 Bellaterra Spain conchi@icmab.es; Departament de Química Inorgànica i Orgànica, Institut de Recerca de Química Teòrica i Computacional, Universitat de Barcelona Diagonal 645 08028 Barcelona Spain; ALBA Synchrotron Light Source 08290 Cerdanyola del Vallés Spain; Instituto de Ciencia y Tecnología del Carbono (INCAR-CSIC) 33011 Oviedo Spain; ICREA, Institució Catalana de Recerca i Estudis Avançats Passeig Lluis Companys 23 08010 Barcelona Spain; Universidad Autónoma de Barcelona, Dept. Química Campus UAB 08193 Bellaterra Spain JoseAntonio.Ayllon@uab.cat

## Abstract

The synthesis of 1D cobalt and zinc monometallic and heterometallic coordination polymers (CPs) was carried out applying one-pot synthetic methods by using either supercritical carbon dioxide or ethanol as the solvent. A collection of four 1D CPs were thus obtained by the combination of a metal (or a mixture of metals) with the linker 1,4-bis(4-pyridylmethyl)benzene. The used metallic complexes were zinc and cobalt hexafluoroacetylacetonate, which can easily incorporate pyridine ligands in the coordination sphere of the metal centre. Independently of the used solvent, the precipitated phases involving Zn(ii), *i.e.*, homometallic CP of Zn(ii) and bimetallic CP of Zn(ii)/Co(ii), were isostructural. Contrarily, homometallic CPs of Co(ii) were precipitated as an isostructural phase of Zn(ii) or with a different structure, depending on the used solvent. All the structures were resolved by XRD using synchrotron radiation. In addition, the magnetic properties of the new CPs involving Co(ii) were studied. Remarkably, at low temperatures with the application of an external field, they acted as field-induced single molecule magnets.

## Abstract 2

One-pot synthesis of heterometallic (Zn(ii)/Co(ii)) nodes directing CP magnetic behaviour to single molecule magnets.

## Graphical abstract

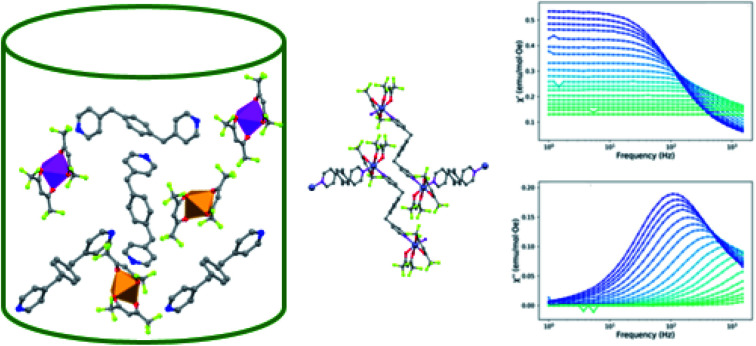

## Introduction

**Fig. 1:**
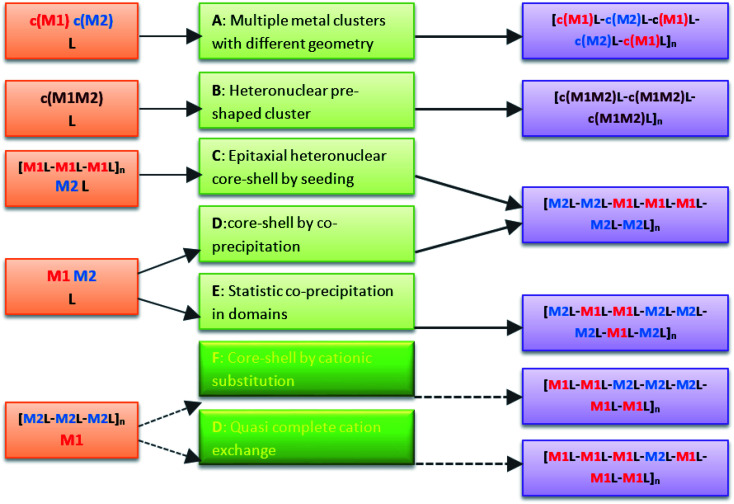
Typical heterometallic frameworks combined with the linker L. Compounds in A and B are built with pre-formed homometallic clusters (c(M1) and c(M2)) or heteronuclear clusters (c(M1M2)), respectively. Compounds in C and D are core–shell structures in which the two homometallic components have lattice match. In C, crystals are precipitated by soaking seed particles of the most stable CP [M1L–M1L–M1L]_*n*_ in a solution of the second metal M2 and the ligand L. In D, sophisticated core–shell MOFs are prepared by co-precipitation of M1/L and M2/L pairs with different nucleation rate, in which the faster precipitating compound (M1L) forms the core and the second precipitates latter as the shell (M2L). Contrarily, in E the statistic co-precipitation of both phases in small domains occurs by using M1/L and M2/L pairs with similar nucleation rate. F and G describe post-synthetic transmetalation, in which crystals of the CP with the lowest stability [M2L–M2L–M2L]_*n*_ are soaked in a solution of the second metal M1 in the absence of ligand. Core–shell structures are obtained at short processing times (F), while almost total replacements can be obtained at high running time (G).

Coordination polymers (CPs) or metal–organic frameworks (MOFs) comprise metal nodes, usually transition metals or metallic clusters, connected in a crystalline network by multipodal organic linkers and coordination bonds. One of the unique features of these extended structures is the tunability in the design, based on the concept of the modification of the composition and geometry of the repeating unit. This includes not only the use of different organic linkers or metal clusters, but also the preparation of multivariate CPs with more than one linker and/or inorganic building unit in the design.^1^ New functionalities and applications are described for these hybrid polymers, particularly in heterogeneous catalysis by designing systems with more than one active center.^2^ One of the earliest synthetic routes described to introduce heterogeneity in CPs was through the organic part, by pre-mixing in the solvothermal synthesis more than one bridging unit. This design is used for compounds involving flexible organic linkers, which are difficult to crystallize in networks with high symmetry factors. The addition of an auxiliary linker, typically a bipyridine or dicarboxylic acid, increases the dimensionality and rigidity of the framework, often providing permanent porosity to the end product.^3^ A second option to introduce heterogeneity in CPs is to use a mixture of metals. Representative heterometallic frameworks are schematized in Fig. 1. Pioneering studies in bimetallic networks concern compounds with several inorganic clusters of different geometries.^4–6^ Next, infinite networks have also been built with heteronuclear nodes involving two metals with different oxidation state.^7–9^ Finally, multilayer core–shell or multidomain single crystals of heterometallic CPs are described for compounds with lattice match.^10–13^ Indeed, the latter has been extended to systems with 10 different divalent metals, possessing a wide range of ionic radii sizes, and a structurally single linker.^14^ Core–shell structures can also be obtained by post-synthetic transmetalation,^15^ a process in which an almost complete exchange of metals ions would be possible by increasing sufficiently the processing time.

Transmetalation is proposed for the preparation of isostructural networks that are difficult to obtain by a direct synthetic strategy, like those involving Ti(iii), V(ii), Cr(ii) and Fe(ii) cations.^16,17^ It is worth mentioning that this is a single crystal-to-single crystal process kinetically viable only for porous compounds with enhanced internal diffusion.^18,19^

Multivariate CPs are usually solvothermally prepared, either in routes of one-pot synthesis or by post-synthetic exchange or cationic substitution. In general, the later can only be used for metals that occupy chemically identical lattice positions, while the former can also be used for systems in which the position of the different cations is not interchangeable. The interest of this study is to demonstrate the feasibility of using one-pot co-precipitation methods, performed in either green supercritical CO_2_ (scCO_2_) or ethanol (EtOH), to develope heterometallic systems. The system chosen for study involves a ditopic bipyridyl derivative, the 1,4-bis(4-pyridylmethyl)benzene (bpymb) linker, and Zn(ii) and/or Co(ii) hexafluoro acetylacetonates as nodes. Zn(ii) and Co(ii) are cations with similar radius, valence and coordination geometry.^20–24^ As a consequence, this two metals have been deeply study to form heterometallic MOFs, in particular those with a zeolitic imidazolate framework, such as ZIF-8 and ZIF-67.^25–28^ In addition, Co(ii) cations are very interesting from the magnetic point of view. In the expected octahedral coordination environment, they have large anisotropy and can behave as single molecule magnets (SMM) or single chain magnets (SCM) in one dimensional systems.^29–34^ In this work, four new CPs were crystallized: one involving Zn(ii), two involving Co(ii) and one involving the mixture of Zn(ii) and Co(ii). The structures of all of them were elucidated by synchrotron X-ray diffraction. For the compounds involving Co(ii), static and dynamic magnetic studies were performed in order to provide insight on their magnetic behavior. Remarkably, homometallic compounds involving Co(ii) and heterometallic Zn(ii)/Co(ii) act as field-induced single molecule magnets (SMMs), expanding this family of appealing systems devoted to the development of high-density information storage materials, quantum computing and molecular spintronic.^35–37^

## Experimental section

### Materials

**Scheme 1:**
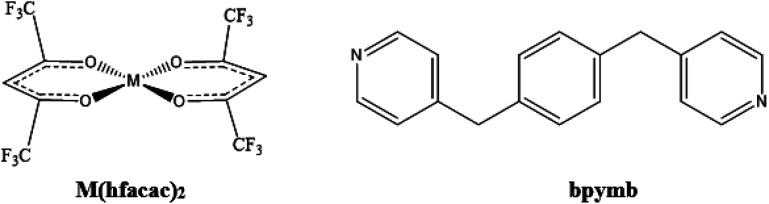
Chemical structures of metal complex M(hfacac)_2_ (M = Zn(ii) or Co(ii)), and the organic linker bypmb.

1,4-Bis(4-pyridylmethyl)benzene (bpymb) was selected as the organic linker and obtained from Cymit. Zinc hexafluoroacetylacetonate dihydrate (Zn(hfacac)_2_·H_2_O) and cobalt hexafluoroacetylacetonate hydrate (Co(hfacac)_2_·*x*H_2_O) were chosen as metal complexes (Scheme 1). These reagents, ethanol (EtOH) and chloroform (CHCl_3_) were all purchased from Sigma Aldrich. Compressed CO_2_ (99.95%) was supplied by Carburos Metálicos S.A.

### Equipment and methods

Crystallization runs were carried out in two different solvents, scCO_2_ and EtOH. Reactions in scCO_2_ were performed in a 5 mL Pyrex vial placed into a 100 mL high pressure autoclave (TharDesign). The vial was charged with *ca.* 100 mg of either Zn(ii) or Co(ii) metal complex and 1 equivalent of bpymb, together with a small magnetic bar. After capping the vial with cellulose paper, scCO_2_ was added up to a pressure of 15 MPa at 333 K, and the system was stirred at 500 rpm. For the precipitation of heterometallic Zn(ii)/Co(ii) CPs, similar conditions as for the single metal compound were used, but with a 0.50 : 0.50 molar ratio in the mixed metal source. After a running period of 3 h, the product was washed with fresh scCO_2_. The autoclave was then depressurized and cooled down to room temperature before recovering a dry powdered sample. For the crystallization tests completed in EtOH, each reagent (*ca.* 100 mg of metal complex and 1 equivalent of bpymb) was separately dissolved in 20 mL of alcohol, then mixed in a 100 mL flask and stirred at 500 rpm for 3 h at room temperature. In all cases, the recovered samples were filtered, rinsed with fresh EtOH and air dried. In some cases, the use of a layering technique was needed to obtain crystals suitable for the single crystal XRD study. The used solutions were obtained by dissolving 4.5 mg of bpymb in 12 mL of EtOH : CHCl_3_ (25 : 75 v%) and 9 mg of metal complex in 2 mL of EtOH. The layering was settled by placing the bpymb solution at the bottom of a vial and slowly adding the metal solution on the top. The vial was then capped and left at room temperature for 1 week to allow the slow liquid–liquid diffusion of one solution into another. Crystals were recovered at the liquid/liquid boundary area and located on the walls.

### Characterization

Routine powder X-ray diffraction (PXRD) patterns of the precipitated compounds were recorded in a Siemens D5000 diffractometer, using the Cu Kα incident radiation in the 2*θ* range of 5 to 30°. Synchrotron single crystal X-ray diffraction (S-SCXRD) experiments for three of the new compounds, namely Zn(1), Co(3) and Zn/Co(4), were performed in the XALOC beamline at the ALBA synchrotron (Spain).^38^ Data were collected at 100 K with a 0.72931 Å wavelength using the Dectris Pilatus 6M detector placed at 120 mm from the sample. The *ϕ*-scans were performed from 0 to 360° in steps of 0.5° and at a collection time of 0.15 s step-1. The scan was repeated at three different *κ* angles (0, 45°, 90°) and merged afterwards to increase the completeness and redundancy when possible. Data were indexed, integrated and scaled using the XDS software.^39^ The crystal structures were solved by intrinsic phasing (SHELXT) and refined with SHELXL (version 2014/7)^40^ using Olex2 as graphical interface.^41^ For the homometallic Co(ii) CP precipitated in scCO_2_ (compound Co(2)) structure elucidation was achieved by synchrotron powder X-ray diffraction (S-PXRD) carried out on a high resolution powder diffraction end station of the MSPD beamline (BL04) at the ALBA synchrotron. Data were collected at 100 and 298 K using the Si-microstrip MYTHEN-II detector (6 modules, 1280 channels per module, 50 μm per channel, sample-to-detector distance 550 mm) at an energy of 25 keV (wavelength = 0.49587 Å determined with the NIST Si standard). Crystallographic data for all compounds are summarized in Tables S1 and S2 in the ESI,† and the corresponding CIF files have been deposited in the Cambridge Crystallographic Data Centre (CCDC 1950459–1950462).

Samples chemical composition was estimated by elemental analysis (E.A., Thermo Carlo Erba Flash 2000). The percentage of each metal in mixed metal structures was determined by inductively coupled plasma mass spectrometry (ICP-MS, Agilent 7700x) after digesting the solid in hydrochloric and nitric acids. Metal distribution was assessed by energy dispersive spectroscopy (EDS, FEI Magellan 400L) in a scanning electron microscopy (SEM, Ultim Extreme Oxford Inst.) with high resolution (0.8–0.9 nm), placing the sample on a holder of silicon wafer and metalizing with Pt. *Ex situ* X ray photoelectron spectroscopy (XPS) was carried out in a XPS Spectrometer Kratos AXIS Supra using Al-Kα monochromatic (20 eV) radiation emitted at 225 W (15 mA/15 kV).

Magnetic measurements were carried out in a Quantum Design SQUID MPMS magnetometer. The analysis was performed on polycrystalline samples of the Co(ii) compounds. Each sample was grinded and introduced in a gelatine capsule that was mounted in a capillary tube made of polyimide for the measurement. Variable temperature magnetic susceptibility measurements between 2 and 300 K were collected under an applied field of 3000 Oe. Reduced magnetization measurements were performed between 1.8 and 6.8 K and applied fields up to 5 T. Dynamic susceptibility measurements under different applied static fields were performed employing an oscillating ac field of 4 Oe in the frequencies range between 1 and 1500 Hz. Diamagnetic corrections of the constituent atoms were estimated using Pascal's tables and the susceptibility was also corrected for the sample holder. In the case of the heterometallic sample Zn(ii)/Co(ii), the acquired data was corrected to have the same *χT* value at 300 K than the Co(ii) isostructural sample precipitated in EtOH, giving a Zn(ii) : Co(ii) atomic ratio of 0.43 : 0.57.

### Computational details

Multireference calculations were performed using two different software packages, OpenMolcas (along with Single Aniso)^42^ and Orca^43^ Neese. The studied systems are CPs, so molecular models, including one Co(ii) ion and the coordinated ligands, were created for each compound from the crystal structure to understand the magnetic properties of the subunits at a molecular level. Employed Cartesian coordinates are on Tables S3–S5.† OpenMolcas version 18.09 and Orca version 4.1.0 version were employed. With the former, the energy of the states was calculated at CASSCF level and then the SO-RASSI (Restricted Active Space State Interaction) approach was employed to mix them and obtain the final energy states.^44^ All electron ANO-RCC basis set were employed: Co (6s 5p 4d 2f), Zn (5s 4p 3d 1f), N (4s 3p 2d 1f), C (3s 2p) and H (2s).^45–47^ In addition, the matrix elements of the transition magnetic moments have been calculated to have an estimation about the probability of transition between two different states of the molecules.^48^ Such matrix elements are calculated as proposed by the golden Fermi rule, as the integral between the two involved states using a magnetic moment operator. For Orca version 4.1.0, CASSCF and NEVPT2 calculations were performed. In this case spin–orbit effects were included using the quasi-degenerate perturbation theory (QDPT) and scalar relativistic effects were taken into account using the DKH (Douglas–Kroll–Hess) procedure. The def2-TZVPP basis set was employed, including the auxiliary basis sets for correlation and Coulomb fitting, for all the atoms.^49,50^ The employed active space in both cases includes seven electrons in the five 3d orbitals of the Co(ii) ion, CAS (7,5). All states for the quartet states arising from the 4F and 4P terms of Co(ii), and all the 40 states for the doublet state coming from the 2P, 2D (x2), 2F, 2G and 2H terms of the Co(ii) ion have been included.

## Results and discussion

### Structural description

**Fig. 2:**
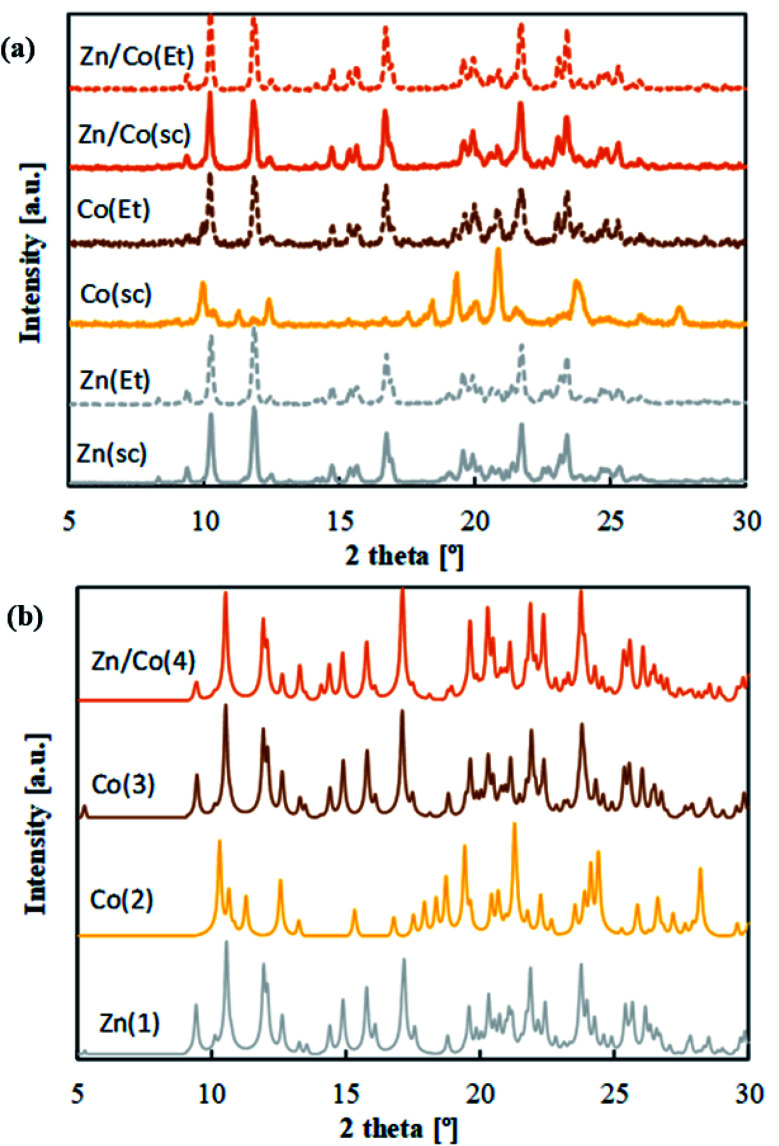
PXRD patterns of: (a) precipitated samples, and (b) simulated from single-crystal data.

Table 1 describes the synthetized mono and bimetallic CPs as a function of processing conditions. Precipitation experiments were performed in scCO_2_ (samples Zn(sc), Co(sc) and Zn/Co(sc)) and EtOH (samples Zn(Et), Co(Et) and Zn/Co(Et)) solvents. Both reagents, M(hfacac)_2_ and bpymb, were highly soluble in these solvents. Hence, precipitation experiments were carried out from solution in both tested solvents. Crystal structures were first assessed by routine PXRD (Fig. 2a). The patterns of samples Zn(sc), Zn(Et), Co(Et), Zn/Co(sc) and Zn/Co(Et) were all similar. On the contrary, the different PXRD pattern displayed by the Co(sc) sample pointed to a dissimilar structure. Differences were also reflected in the observed color of the precipitated samples. Any compound involving only Zn(ii) exhibits a white color, while the products with pure Co(ii) reflect either an orange color for Co(sc) or orange-brown for Co(Et). This last color was also observed for the Zn/Co(sc) and Zn/Co(Et) samples. By using the layering method in EtOH, single crystals were successfully produced for the S-SCXRD structure elucidation of samples Zn(Et) (and, by extension, Zn(sc)), Co(Et) and Zn/Co(Et) (and, by extension, Zn/Co(sc)), denoted as compounds Zn(1), Co(3) and Zn/Co(4). The unidentified structure of Co(sc) was elucidated from the powder by S-PXRD techniques (compound Co(2)).

**Table 1:** Solvents of synthesis and composition of the different precipitated compounds

Sample	Solvent	Compound^a^	Crystal structure	E.A. (C, H, N) [%wt]
Zn (sc)	scCO_2_	*cis*-[Zn(hfaac)_2_(bpymb)]_*n*_, Zn(1)	A	f. 45.52, 2.43, 3.88
Zn (Et)	EtOH			c. 45.46, 2.45, 3.79
Co(sc)	scCO_2_	*trans*-[Co(hfaac)_2_(bpymb)]_*n*_, Co(2)	B	f. 45.71, 2.36, 3.94
				c. 45.86, 2.47, 3.82
Co(Et)	EtOH	*cis*-[Co(hfaac)_2_(bpymb)]_*n*_, Co(3)	A	f. 45.69, 2.36, 3.91
				c. 45.86, 2.47, 3.82
Zn/Co(sc)	scCO_2_	*cis*-[Zn_0.5_Co_0.5_(hfaac)_2_(bpymb)], Zn/Co(4)	A	f. 45.7, 2.36, 3.94
Zn/Co(Et)	EtOH			c. 45.66, 2.46, 3.80

a The *cis/trans* prefix make reference to the relative position of the N pyridine atoms around the metallic center.

**Fig. 3:**
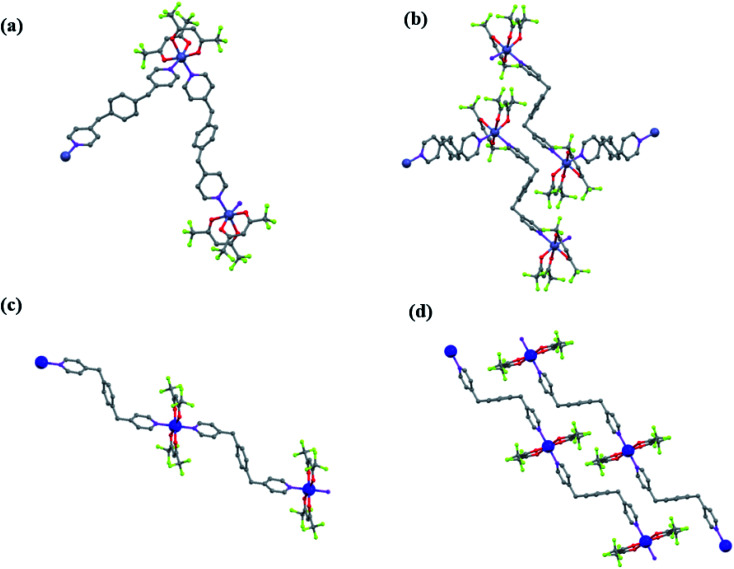
Schematic representation of structures: (a and b) A (Zn(1), Co(3), Zn/Co(4)), and (c and d) B, Co(2). Color legend: Zn or Co, blue; C, grey; O, red; N, violet; F, violet; H are omitted for clarity.

The structures of these compounds are described grouped in the two isostructural frameworks (structures A and B in Fig. 3).

#### Structure A

Zn(1), Co(3) and Zn/Co(4) crystallize in the triclinic *P*1̄ space group. The crystallographic angles were very similar for the three products, with values between *α* = 104.4–104.7°, *β* = 96.0–96.4° and *γ* = 97.4–97.5°. For this structure, the asymmetric unit involves an hexacoordinated metal linked to two bpymb (half) molecules and two hfacac groups (Fig. 3a), thus giving the stoichiometry [M(hfacac)_2_(bpymb)]_*n*_. The two bpymb linkers are located in relative *cis* positions, forming N–M–N angles of 93.7, 92.8 and 93.1° for Zn(1), Co(3) and Zn/Co(4), respectively. As a consequence, the structure extends in zig-zag chains. For all compounds, F⋯H–C_ar_ and F⋯F weak interactions are established among chains, resulting in stable 3D crystals (Fig. 3b). Despite the larger covalent radii of Co(ii) (1.26 Å) *vs.* Zn(ii) (1.22 Å),^51^ the unit cell volumes of the Co(3) and Zn(1) compounds were the same (1464 Å^3^). Contrarily, the heterometallic compound Zn/Co(4) displayed a significant larger value (1471 Å^3^) than the homometallic counterparts, likely originated by the slim distortion introduced in the network by the presence of two metals with distinct radii.

#### Structure B

Co(2) crystallizes in the triclinic *P*1̄ space group. As occurs for the three isostructural compounds with framework A, the asymmetric unit involves hexacoordinated Co(ii) linked to two bpymb ligands and two hfacac groups (Fig. 3c), with a stoichiometry of [M(hfacac)_2_(bpymb)]_n_. However, in this case the two bpymb linkers are located in a *trans* positions, with an N–Co–N angle of 180.0°. The polymer extends in 1D chains that interact through F⋯F intermolecular weak bonds (Fig. 3d).

Comparing Co(2) and Co(3) crystal structures with frameworks B and A, respectively, it becomes evident that the structural changes are mainly originated on the large differences of the values of the angle N–Co–N, of 180.0° for Co(2) and almost half of this value (92.8°) for Co(3). The rest of the structure is similar, since in both cases the bpymb linker adopts a Z configuration and the coordination of the Co(ii) is completed with two hfacac units. This change in the N–Co(ii)–N angle also induces a noticeable variation of the density of the crystal, larger (1.788 g cm^−3^) in Co(2) than in Co(3) (1.664 g cm^−3^). In both structures the shortest metal–metal distances are the intermolecular (*ca.* 9 Å), while an intra-chain metal–metal distances of *ca.* 16 Å.

### Composition and spatial arrangement of Zn(ii) and Co(ii) in mixed metal CPs

Phase purity of the different precipitated compounds was confirmed by comparison of routine PXRD patterns and simulated profiles calculated from data of the single-crystal structures (Fig. 2a and b). E.A. data also corroborate a high degree of purity in the precipitated samples, since the C, N, H values calculated for the stoichiometry [M(hfacac)_2_bpymb]_*n*_ match the experimentally found values (Table 1). Quantification of the two metals in the heterometallic compound was performed by ICP-MS in several samples of the Zn/Co(4) phase prepared in either EtOH or scCO_2_. The starting molar ratio of Zn(ii) : Co(ii) metals was always 0.50 : 0.50. The measured molar (or equivalent atomic) percentages were in the order of 0.50 ± 0.10 for both Zn(ii) and Co(ii), *i.e.*, the molar ratio values were close to the added amount of each metal.

The preparation of the heterometallic CP produces at all times the Zn/Co(4) phase with framework A, indicating that this structure is the thermodynamically most stable, even the phases involving pure Co(ii) might crystallize in different polymorphic crystal lattices. This fact was confirmed in an extra experiment performed in scCO_2_ with an initial molar ratio Zn(ii) : Co(ii) of 0.2 : 0.8. The product was an heterometallic compound with stoichiometry [Zn_0.15_Co_0.85_(hfacac)_2_bpymb]_*n*_ and A-type framework. Hence, even a small amount of Zn(ii) present in the Co(ii) scCO_2_ solution directs the crystallization to the structure A instead of B, which is a framework exclusively found for homometallic Co(ii) in this solvent. It should be taken into account that only the mixture of Zn(1) and Co(3), both with framework A, produces a precipitate with lattice match, not possible for the mixture of Zn(1) and Co(2) with frameworks A and B, respectively. One additional advantage of the co-precipitation method is that the metal content of the resulting heterometallic compound can be controlled through variations in the molar ratio of the precursor mixture.

**Fig. 4:**
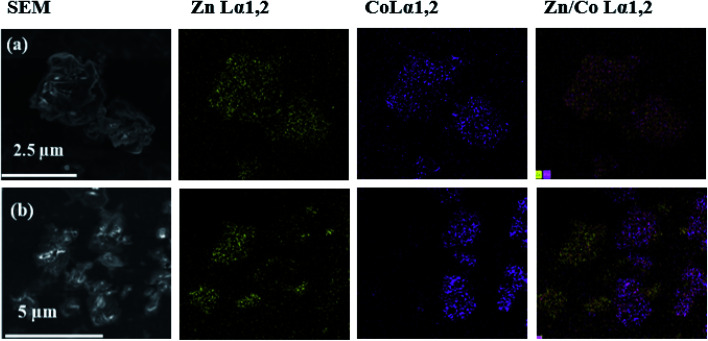
EDS mapping of: (a) Zn/Co(sc) sample, and (b) physical mixture of Zn(sc) and Co(Et) in a molar ratio 0.50 : 0.50.

EDS general spectra with nanometric resolution were acquired at different spots of the SEM images of the heterometallic compound to investigate metal distribution. Elemental mapping for the Zn/Co(sc) sample are here described as an illustrative example of the Zn/Co(4) compound (Fig. 4a). For comparison, a physical mixture was obtained by integrating powder of the isostructural Zn(sc) and Co(Et) samples (Fig. 4b). The mapping of Zn/Co(sc) shows an identical distribution of Zn(ii) and Co(ii) in the analyzed sections, which suggests that both ions were evenly spread throughout the particles. The EDS gives an approximated atomic ratio of Zn(ii) : Co(ii) of 0.51 : 0.49. Contrarily, the physical mixture displayed Zn(ii) and Co(ii) segregated in spots representing the different homometallic specimens. Additionally, the EDS spectrum obtained for one individual crystal of the sample Zn/Co(sc) indicates again equal and homogeneous distribution of both metals, showing proper intermixing of Zn(ii) and Co(ii) atoms (Fig. S1†). It is worth pointing that EDS has a nanometric resolution. Therefore, this technique is only indicating that the Zn(ii) and Co(ii) atoms were evenly scattered around the sample at a nanometric level. However, they can be distributed in the framework either as intercalated metals or segregated into small (smaller than the EDS resolution) domains of homometallic CPs. This behavior has been theoretically demonstrated for other heterometallic MOFs involving Mg/Ni and Mg/Cd.^52^

### Surface analysis

XPS is a powerful tool to collate information on the chemical compositions of materials surface, their coordination chemistry and degradation behavior. Moreover, the technique has also been proposed to help on the detection of the spatial position of the different metals in heterometallic CPs. For compounds with an even distribution of metals, the coordination environment of each cation would be influenced by the presence of adjacent metals of different characteristics. Theoretically, significant shifts in the binding energies would be observed in the mixed metal compound compared to the corresponding isostructural homometallic products. However, for the samples studied in this work no shift in the binding energy of the metals in Zn/Co(4) was observed with respect to Zn(1) and Co(3) (Fig. S2†). The clearest spectrum was the one obtained for the Zn(sc) sample, in which the low binding energy peak in the Zn2p3/2 region was placed at 1021.9 eV. The same peak for the Zn/Co(sc) sample appeared at 1022.1 eV, *i.e.*, with a negligible binding energy shift. The deconvolutions peaks for the Co 2p_3/2_ sub-region were also similar in the Co(Et) and Zn/Co(sc) sample. The conclusion is that the interaction between the two different metals was not significant for XPS detection. Again, this technique cannot discern between structures obtained by metals intercalation or homometallic domains. The analysis of the total amount of Zn(ii) and Co(ii) in the Zn/Co(sc) sample indicated a surface rich in Co(ii), with a Zn(ii) : Co(ii) molar ratio of 0.38 : 0.62. In scCO_2_, this fact can reflect the faster nucleation of the Zn(ii) phase with respect to the isostructural Co(ii) with framework A, since in this fluid the most stable phase for homometallic Co(ii) solutions was the one with framework B.

For the compounds synthetized in this work, an evident change of color was observed as a function of the XPS measurement time, which was as clear indication of the degradation process provoked by the X-ray radiation.^53^ A quantitative analysis performed on the basis of the percentage of the different atoms in each sample (Table S6†) allows to estimate that the percentage of degradation was higher than 50 mol% for the three studied samples Zn(sc), Co(Et) and Zn/Co(sc). This fact makes difficult to mine extra information from XPS data.

### Magnetic properties

CPs formed by Co(ii) subunits are good candidates as single chain magnets (SCMs)^54,55^ or single molecule magnets (SMMs)^56,57^ due to the intrinsic magnetic anisotropy of the Co(ii) in various geometries, including the pseudo-octahedral,^58–61^ and the nature of the resulting exchange interaction through their linkers. The studied systems have a small distortion with respect to the ideal octahedral geometry as showed by their shape measurement, 0.059, 0.465 and 0.520 for Co(2), Co(3) and Zn/Co(4) respectively. Herein, the magnetic properties of the reported new CPs are explored.

**Fig. 5:**
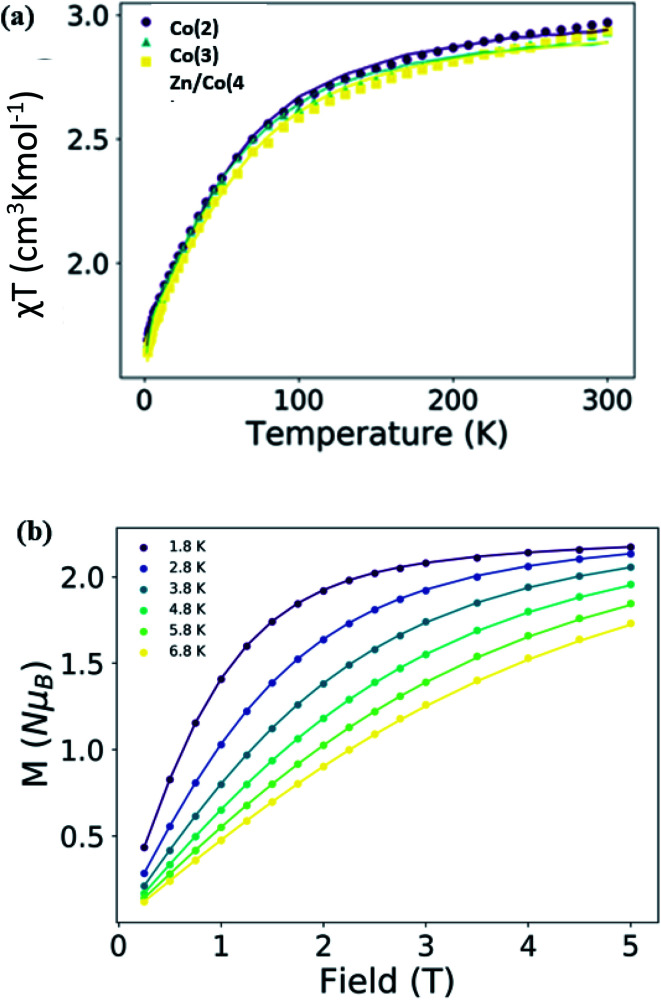
(a) *χT vs.* temperature curve for compounds Co(2), Co(3) and Zn/Co(4) at an applied field of 3000 Oe, and (b) *M vs.* field curves at different temperatures for Co(2). The lines are the results of the fit with PHI package.

The variable temperature susceptibility and magnetization curves of Co(2), Co(3) and Zn/Co(4) are similar and depicted in Fig. 5, S3 and S4.† Despite the structural differences among them, the magnetic properties are almost identical, highlighting the similar environment and isolation of the Co(ii) units in the systems. At room temperature, the *χT* product is close to 3 cm^3^ K mol^−1^ in all of the cases, larger than the expected for a *S* = 3/2 orbitally non-degenerate ground state (1.875 cm^3^ K mol^−1^). This indicates that the systems have a large unquenched orbital contribution, which is characteristic of Co(ii) ions in pseudo-octahedral coordination.^59,60,62–67^

When lowering temperature, the *χT* product slowly decreases to the minimum of 1.64 cm^3^ K mol^−1^ observed at 2 K, which is mainly due to spin orbit coupling (SOC). For Co(ii) ions in a distorted octahedral geometry the first order SOC would be important and the experimental data have been sometimes fitted with the corresponding Hamiltonian. However, the large energy difference between the states implies that only the lowest Kramers doublets are populated and, phenomenologically, the zero-field splitting (ZFS) Hamiltonian can be used to study the magnetic properties of this compounds.^67–71^ Herein, we employed the later approach for sake of simplicity and to allow further comparison with published systems.^58,60–67^ This way, the ZFS Hamiltonian, as implemented in the PHI package (eqn (1)), was employed for the simultaneous fit of the experimental susceptibility and magnetization curves.1
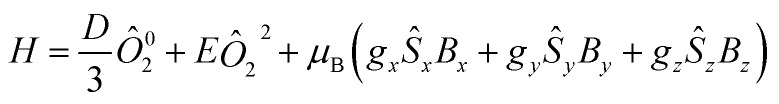
where *S* is the spin (and their spin operators), *D* is the axial anisotropy, *E* the rhombic anisotropy, *μ*_B_ is the Bohr magneton, *B* is the magnetic field vector and *Ô*_*k*_^*q*^ is the equivalent Stevens operator. The first two terms relate to the crystal-field Hamiltonian (following the operator equivalent technique), and the last term is connected to the Zeeman Hamiltonian. In addition, due to the polymeric character of the compounds, the possible antiferromagnetic interactions between the subunits should be considered despite the long size of the linker. This possible effect has been included in the fit of the magnetic data by applying the van Vleck equation in the mean field approximation as implemented in the PHI package.^72^

The results of the fit, Table 2 and Fig. 5, show that this kind of compounds has easy plane anisotropy with a positive and relatively large, around 50 cm^−1^, *D* values and with two *g*_i_ components larger than *g*_e_. Besides the 1D nature of the compounds the interaction between units is very small with no significant difference between the magnetically diluted sample Zn/Co(4) and the others. This indicates that the linker effectively separates the magnetic subunits and no SCM behavior would be expected, only SMM.^73–76^ The observed behavior and anisotropic values are similar to other published mononuclear Co(ii) complexes in pseudo-octahedral geometry and also in other Co(ii) CPs.^77–82^

**Table 2:** Results from the fitting of compounds Co(2), Co(3) and Zn/Co(4), including *D*, *E*, *g*_*x*_, *g*_*y*_, *g*_*z*_ and *zJ* parameters

	Co(2)	Co(3)	Zn/Co(4)	Confidence interval
*D* (cm^−1^)	46.3	48.0	55.1	±0.4
*E* (cm^−1^)	9.4	11.4	14.0	±0.2
*g*_*x*_	1.73	1.86	2.09	±0.02
*g*_*y*_	2.60	2.58	2.51	±0.01
*g*_*z*_	3.13	3.00	2.91	±0.01
*zJ* (cm^−1^)	−0.012	−0.017	−0.019	±0.001

**Fig. 6:**
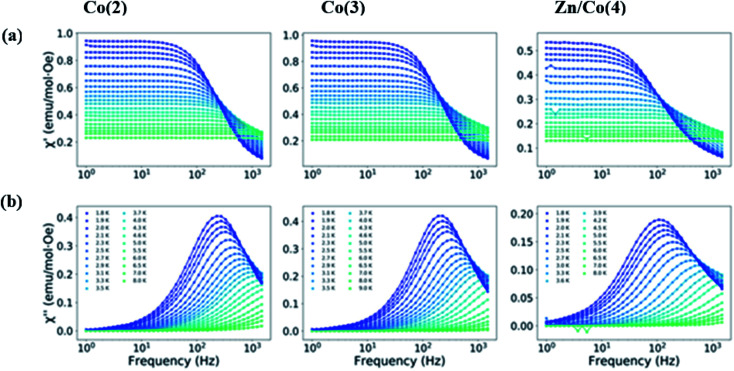
Frequency dependence of: (a) real and (b) imaginary parts of the magnetic susceptibility at an applied static dc field of 1500 Oe and for different temperatures for compounds Co(2), Co(3) and Zn/Co(4). The lines are a guide for the eye.

Dynamic susceptibility measurements were performed to study the possibility of slow relaxation of the magnetization. Without an applied static dc field, no *χ*′ signal was observed; however, when an external dc field was used, a dependence of *χ*′ appeared. Therefore, the dependence with the field for the different compounds was studied (Fig. S5–S7†). Under the latest conditions, a maximum in *χ*′ appeared around 500 Hz for the three different compounds indicating that they all behave as field induced mononuclear SMMs. The maximum slightly moves to higher frequencies when the applied dc field was increased. A common static dc field of 1500 Oe was selected for the study of the temperature dependence of the dynamic susceptibility for the three compounds (Fig. 6). Similar frequency dependence of the out of phase component of the susceptibility was observed for both Co(2) and Co(3) despite their different geometry. However, for the magnetically diluted sample Zn/Co(4) the maximum in *χ*′ moves to lower frequencies, which might be due to the decrease of dipolar interactions. This result indicates that, although the distance between paramagnetic centers was already large, and the magnetic dilution stoichiometry still leaves several nearest neighbors (assuming a random distribution of the metal ions), part of the dipolar interactions between Co(ii) centers are being reduced.

The Cole–Cole plots were fitted to a generalized Debye model using CCfit package (Fig. S8–S10 and Tables S7–S9†). Temperatures from 1.8 to 9 K were analyzed, however for temperatures larger than 4.6 K less than half semicircles were observed in the Cole–Cole plot and consequently that data was not included in the analysis. The obtained *α* values were always smaller than 0.16, indicating a narrow distribution of relaxation times.

Several different relaxation processes are usually proposed for mononuclear SMMs.^60,83^ The Arrhenius expression (eqn (2)) was employed to fit the high temperature region of the analyzed data to account for a thermally activated process, the so-called Orbach process. The obtained energy barriers are similar for the studied compounds and they are found in the range of 5.9 and 11.3 cm^−1^, with *τ*_0_ values in the order of 10^−5^ to 10^−6^. It should be noticed that the energy barrier should correspond to the energy difference between the ground and first excited doublet states. That energy difference can be estimated from the *D* values obtained in the simultaneous fit of the susceptibility and magnetization (*vide supra*) and from *ab initio* calculations (*vide infra*). In both cases the energy difference between those states is larger than the obtained here. It indicates that the relaxation of the spin is not occurring *via* an Orbach process and other process or processes are the main responsible.

2 *τ*^−1^ = *τ*_0_^−1^*e*^−*U*_eff_/*k*_*B*_*T*^

**Fig. 7:**
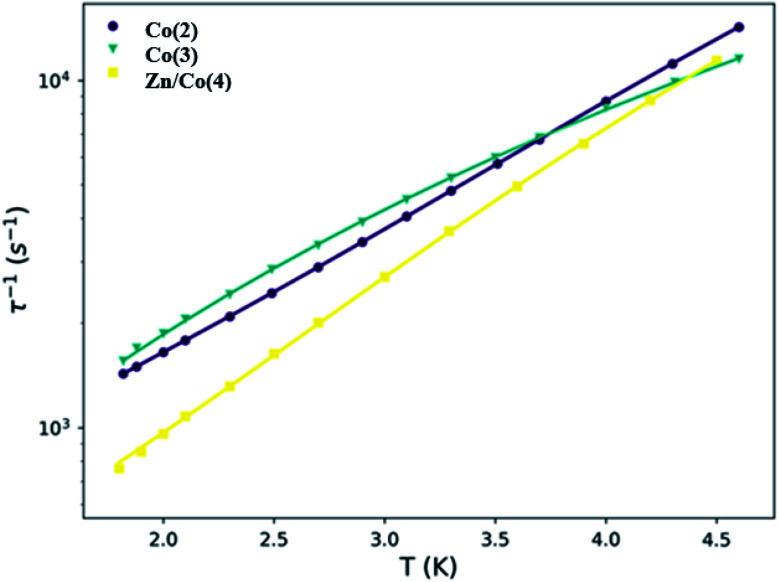
Temperature dependence of the relaxation time for compounds Co(2), Co(3) and Zn/Co(4). The solid lines are the result of the fit with eqn (3).

In this regard, the principal spin relaxation processes are the quantum tunneling of the magnetization (QTM), direct and Raman processes. All of them have been considered for the fit of the dependence of the relaxation time with temperature in eqn (3), Table 3 and Fig. 7. The first term accounts for the direct term, with a linear dependence with temperature, the second corresponds to the temperature independent QTM term and the last one describes the Raman term, with a temperature *n* exponent that can have different values depending on the system and the available phonons.^60,83^

3 *τ*^−1^ = *AH*^4^*T* + *B* + *CT*^*n*^

**Table 3:** Fitting parameters obtained from the fit of the relaxation time using eqn (3)

	Co(2)	Co(3)	Zn/Co(4)
*A* (s^−1^ K^−1^)	9.57 × 10^5^	1.35 × 10^6^	6.67 × 10^5^
*B* (s^−1^)	8.8 × 10^−10^	0	0
*C* (s^−1^ K^−*n*^)	124.5	12.8	14.4
*N*	2.83	4.42	4.34

In all three cases, the QTM is not responsible for the relaxation process because of the applied static dc field. The direct process leads the spin relaxation at low temperatures while the Raman process does it at higher temperatures. Despite the similitude between Co(2) and Co(3) curves, the results of the fit are different, especially in the Raman process; the pre-exponential term decreases one order of magnitude from Co(2) to Co(3), while the exponent increases. The exponent for Kramers ions should be 9, however values between 1 and 6 are usually observed and accepted in this type of compounds and attributed to the presence of acoustic and optical phonons.^84^ The differences between Co(2) and Co(3) curves may relate to the different structure and packing. The main differences between Co(3) and Zn/Co(4) are observed at low temperature, where the direct process is the predominant. The smaller *A* value for Zn/Co(4) can be attributed to the magnetic dilution and the decrease of dipolar interactions. Overall, the systems have similar magnetic behaviors; the discrepancy found in some of the parameters may relate to the magnetic dilution and the lower concentration of the Co(ii) ions due to the employed ratio and despite the non-homogeneous distribution indicated by XPS of the Zn/Co ions in the samples.

**Fig. 8:**
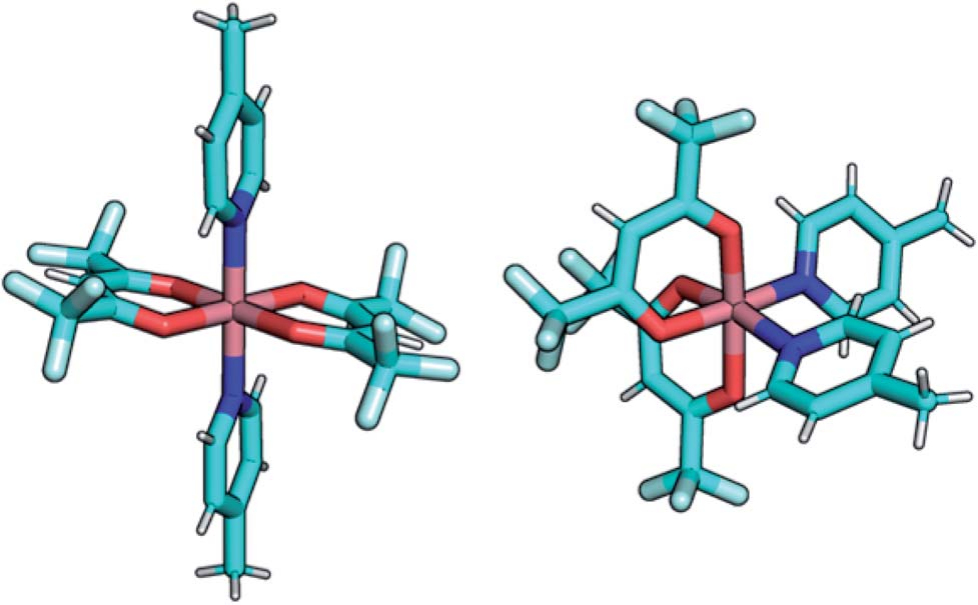
Graphical representation of the employed models in the multireference calculations, compounds Co(2) and Co(3), left and right, respectively.

To gain insight on the magnetic properties of the studied compounds, multireference calculations using OpenMolcas and ORCA software packages (see Computational details) were carried out. A molecular model was employed for all the compounds and the geometries obtained from the crystal structures were used, where the bridging ligand bpymb was modelled as 4-methylpyridine. Fig. 8 shows the employed models for compounds Co(2) and Co(3). For Co(ii) complexes, the dynamic correlation can be relevant so CASPT2 results are shown in the manuscript while CASSCF are in the ESI (Table S10†).

In the past, some of us demonstrated that octahedral Co(ii) compounds can present positive or negative large *D* values depending on the splitting of the d orbitals by the crystal field.^66^ Our predictions were based on the analysis of the *D*_ii_ components of the tensor. The qualitative model shows that the *D* value depends on the orbitals involved in the excitation and the energy different between them. This way, small energy differences results into large *D* values. If the orbitals involved in the first excitation have the same |*m*_*l*_| value, then *D* will be negative, as the largest contribution will be *D*_zz_. On the contrary, when the orbitals involved in the first excitation have a *m*_*l*_ change of ±1, then the largest contributions will be *D*_*xx*_ and *D*_*yy*_ and *D* will be positive.^66,85^ The main calculated parameters are collected in Table 4. It can be observed that the first excitation energy before SOC is small, which gives to a large energy difference between the ground and first excited KDs. In addition, the ground state *g*_i_ factors indicates an easy plane character that, in some cases, is closer to an intermediate situation between easy plane and easy axes, providing three *g*_i_ factor values larger than 2 and ranging between 2.1 and 6.6. The large *D* and *E* values and the *g*_i_ factors of the ground state allow us to describe the system as rhombic anisotropy. Although the excited state in all the cases is axial. When comparing with the experimental values obtained from the simultaneous fit of the susceptibility and magnetization data can be observed that the anisotropy of this compounds with a relatively large *D* and *E* values is well described. However, the *D* and *g*_i_ values are overestimated, probably due to the intrinsic limitations of the calculations and the created molecular model. Although it is worth mentioning that the comparison of the susceptibility and magnetization curves obtained from the CASPT2 calculations with the experimental ones matches well (Fig. S11†).

**Table 4:** Calculated first excitation energies with (*δE*_1_) and without (Δ*E*_1_) spin–orbit coupling (cm^−1^) and ZFS parameters (*D* and *E* in cm^−1^) and g-factors of the ground and first excited states for the studied molecules at CASPT2 (NEVPT2) level

		Co(2)	Co(3)	Zn/Co(4)
Δ*E*_1_ (cm^−1^)		302 (374)	680 (591)	699 (531)
δ*E*_1_ (cm^−1^)		215 (204)	137 (165)	141 (180)
*D* (cm^−1^)		106.2 (99.3)	65.4 (75.8)	66.8 (81.0)
|*E*| (cm^−1^)		10.3 (12.9)	11.4 (18.6)	12.8 (22.4)
KD_1_	*g*_*x*_	5.48 (2.60)	2.23 (1.99)	2.15 (1.93)
	*g*_*y*_	4.31 (3.74)	3.50 (2.91)	3.37 (2.66)
	*g*_*z*_	3.03 (6.31)	6.44 (7.13)	6.58 (7.41)
KD_2_	*g*_*x*_	0.40 (0.98)	1.23 (1.76)	1.33 (1.91)
	*g*_*y*_	0.52 (1.17)	1.33 (1.89)	1.45 (2.15)
	*g*_*z*_	5.40 (5.39)	5.87 (5.48)	5.79 (5.28)

**Fig. 9:**
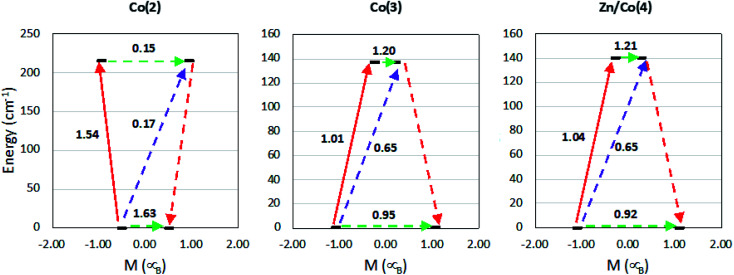
Calculated states energies as function of their average projected magnetic moment for the first and second KDs and transition probabilities between states. Values above 0.1 indicate an efficient spin relaxation mechanism. The dashed green arrows correspond to the tunneling process, dashed purple arrow shows the hypothetical Orbach relaxation process and the red arrows indicate the transition between the ground and excited Kramer's doublets (solid) and the relaxation pathway to the ground state with the reversed spin (dashed).

The different transition probabilities have been computed and are shown in Fig. 9. In all the cases, the transition probabilities of both tunneling and Orbach processes are large. The large tunneling probabilities rationalize the absence of SMM behavior at zero field in this family of compounds. Once an external static field is applied, an energy barrier of more than 140 cm^−1^ is expected due to the also large transition probabilities obtained. However, the experimental values do not present such pattern. This is common in mononuclear complexes due to the presence other relaxation processes not included in the calculations. Those indicate that computational results agree with experimental data, rationalizing the anisotropy of these CPs. The easy plane anisotropy is confirmed; however, as it is seen from the fit of the dynamic susceptibility measurements, additional relaxation processes, such as Raman and direct terms, are the responsible of the observed spin relaxation at the lowest temperatures.

## Conclusions

Multivariate CPs involving two different metals (*i.e.*, Zn(ii) and Co(ii), with similar radius and equal valence) and a ditopic bipyridyl derivative linker were prepared by a one-pot co-precipitation method performed in either scCO_2_ or EtOH solvent. Four new CPs were thus crystallized: one homometallic involving Zn(ii), two homometallic involving Co(ii) and one heterometallic involving the mixture of Zn(ii) and Co(ii). Synchrotron XRD characterization was used to elucidate the crystal structures, but could not discriminate between both metals in the framework. EDS and XPS analysis indicated that the metals were evenly mixed along the heterometallic sample. Static and dynamic magnetic studies were performed in order to provide insight on the magnetic behavior of Co(ii) compounds linked to metal distribution in the structure. For the three CPs involving Co(ii), despite the structural differences, the magnetic properties were almost identical, highlighting the similar environment and isolation of the Co(ii) units in each system. The used linker effectively separates the magnetic subunits and no single chain magnet behavior was observed. However, homometallic compounds with Co(ii) and heterometallic Zn(ii)/Co(ii) act as field-induced single molecular magnets.

## Conflicts of interest

There are no conflicts to declare.

## Author contributions

Conceptualization: C. Domingo, J. A. Ayllón; funding acquisition: C. Domingo, A. López-Periago, N. Aliaga; Investigation: N. Portolés, A. M. López; formal analysis: O. Valcorba, S. Gómez-Coca, G. Marbán; project administration: C. Domingo, N. Aliaga-Alcalde; writing-review & editing: C. Domingo, J. A. Ayllón, N. Aliaga-Alcalde, S. Gómez-Coca.

## Funding sources

Ministerio de Ciencia, Innovación y Universidades; Generalitat de Catalunya.

## Supplementary Material

RA-010-D0RA09132D-s001

RA-010-D0RA09132D-s002

## References

[cit1] Furukawa H., Müller U., Yaghi O. M. (2015). “Heterogeneity within Order” in Metal–Organic Frameworks. Angew. Chem., Int. Ed..

[cit2] Dhakshinamoorthy A., Asiri A. M., Garcia H. (2016). Mixed-metal or mixed-linker metal organic frameworks as heterogeneous catalysts. Catal. Sci. Technol..

[cit3] Bunck D. N., Dichtel W. R. (2013). Mixed Linker Strategies for Organic Framework Functionalization. Chem.–Eur. J..

[cit4] Tu B., Pang Q., Ning E., Yan W., Qi Y., Wu D., Li Q. (2015). Heterogeneity within a Mesoporous Metal–Organic Framework with Three Distinct Metal-Containing Building Units. J. Am. Chem. Soc..

[cit5] Ghoshal D., Ghosh A. K., Maji T. K., Ribas J., Mostafa G., Zangrando E., Ray Chaudhuri N. (2006). Different topologies in heterometallic frameworks of copper(ii) with bridging ligand: Syntheses, crystal structures, thermal and magnetic properties. Inorg. Chim. Acta.

[cit6] Yuan S., Qin J.-S., Li J., Huang L., Feng L., Fang Y., Lollar C., Pang J., Zhang L., Sun D., Alsalme A., Cagin T., Zhou H.-C. (2018). Retrosynthesis of multi-component metal–organic frameworks. Nat. Commun..

[cit7] Wang Y., Bredenkötter B., Rieger B., Volkmer D. (2007). Two-dimensional metal–organic frameworks (MOFs) constructed from heterotrinuclear coordination units and 4,4′-biphenyldicarboxylate ligands. Dalton Trans..

[cit8] Zhang J.-P., Ghosh S. K., Lin J.-B., Kitagawa S. (2009). New Heterometallic Carboxylate Frameworks: Synthesis, Structure, Robustness, Flexibility, and Porosity. Inorg. Chem..

[cit9] Zhang Y., Chen B., Fronczek F. R., Maverick A. W. (2008). A nanoporous Ag–Fe mixed-metal–organic framework exhibiting single-crystal-to-single-crystal transformations upon guest exchange. Inorg. Chem..

[cit10] Furukawa S., Hirai K., Nakagawa K., Takashima Y., Matsuda R., Tsuruoka T., Kondo M., Haruki R., Tanaka D., Sakamoto H., Shimomura S., Sakata O., Kitagawa S. (2009). Heterogeneously Hybridized Porous Coordination Polymer Crystals: Fabrication of Heterometallic Core–Shell Single Crystals with an In-Plane Rotational Epitaxial Relationship. Angew. Chem., Int. Ed..

[cit11] Lee H. J., Cho Y. J., Cho W., Oh M. (2013). Controlled Isotropic or Anisotropic Nanoscale Growth of Coordination Polymers: Formation of Hybrid Coordination Polymer Particles. ACS Nano.

[cit12] Sibille R., Mazet T., Malaman B., Wang Q., Didelot E., François M. (2015). Site-Dependent Substitutions in Mixed-Metal Metal–Organic Frameworks: A Case Study and Guidelines for Analogous Systems. Chem. Mater..

[cit13] Tanasaro T., Adpakpang K., Ittisanronnachai S., Faungnawakij K., Butburee T., Wannapaiboon S., Ogawa M., Bureekaew S. (2018). Control of Polymorphism of Metal–Organic Frameworks Using Mixed-Metal Approach. Cryst. Growth Des..

[cit14] Wang L. J., Deng H., Furukawa H., Gándara F., Cordova K. E., Peri D., Yaghi O. M. (2014). Synthesis and Characterization of Metal–Organic
Framework-74 Containing 2, 4, 6, 8, and 10 Different Metals. Inorg. Chem..

[cit15] Carson C. G., Ward J., Liu X. T., Schwartz J., Gerhardt R. A., Tannenbaum R. (2012). Dopant-Controlled Crystallization in Metal–Organic Frameworks: The Role of Copper(ii) in Zinc 1,4-Benzenedicarboxylate. J. Phys. Chem. C.

[cit16] Kim M., Cahill J. F., Fei H., Prather K. A., Cohen S. M. (2012). Postsynthetic Ligand and Cation Exchange in Robust Metal–Organic Frameworks. J. Am. Chem. Soc..

[cit17] Brozek C. K., Dincă M. (2013). Ti 3+ -, V2+/+3, Cr 2+/3, Mn 2+ , and Fe 2+ -Substituted MOF-5 and Redox Reactivity in Cr- and Fe-MOF-5. J. Am. Chem. Soc..

[cit18] Das S., Kim H., Kim K. (2009). Metathesis in Single Crystal: Complete and Reversible Exchange of Metal Ions Constituting the Frameworks of Metal–Organic Frameworks. J. Am. Chem. Soc..

[cit19] Hon Lau C., Babarao R., Hill M. R. (2013). A route to drastic increase of CO_2_ uptake in Zr metal organic framework UiO-66. Chem. Commun..

[cit20] Song X., Kim T. K., Kim H., Kim D., Jeong S., Moon H. R., Lah M. S. (2012). Post-Synthetic Modifications of Framework Metal Ions in Isostructural Metal–Organic Frameworks: Core–Shell Heterostructures *via* Selective Transmetalations. Chem. Mater..

[cit21] Song X., Jeong S., Kim D., Lah M. S. (2012). Transmetalations in two metal–organic frameworks with different framework flexibilities: Kinetics and core–shell heterostructure. CrystEngComm.

[cit22] Lee W. R., Ryu D. W., Phang W. J., Park J. H., Hong C. S. (2012). Charge effect of foreign metal ions and the crystal growth process in hybridized metal–organic frameworks. Chem. Commun..

[cit23] Fei H., Cahill J. F., Prather K. A., Cohen S. M. (2013). Tandem Postsynthetic Metal Ion and Ligand Exchange in Zeolitic Imidazolate Frameworks. Inorg. Chem..

[cit24] Sun D., Sun F., Deng X., Li Z. (2015). Mixed-Metal Strategy on Metal–Organic Frameworks (MOFs) for Functionalities Expansion: Co Substitution Induces Aerobic Oxidation of Cyclohexene over Inactive Ni-MOF-74. Inorg. Chem..

[cit25] Tang J., Salunkhe R. R., Liu J., Torad N. L., Imura M., Furukawa S., Yamauchi Y. (2015). Thermal Conversion of Core–Shell Metal–Organic Frameworks: A New Method for Selectively Functionalized Nanoporous Hybrid Carbon. J. Am. Chem. Soc..

[cit26] Chen Y.-Z., Wang C., Wu Z.-Y., Xiong Y., Xu Q., Yu S.-H., Jiang H.-L. (2015). From Bimetallic Metal–Organic Framework to Porous Carbon: High Surface Area and Multicomponent Active Dopants for Excellent Electrocatalysis. Adv. Mater..

[cit27] Saliba D., Ammar M., Rammal M., Al-Ghoul M., Hmadeh M. (2018). Crystal Growth of ZIF-8, ZIF-67, and Their Mixed-Metal Derivatives. J. Am. Chem. Soc..

[cit28] Hillman F., Zimmerman J. M., Paek S.-M., Hamid M. R. A., Lim W. T., Jeong H.-K. (2017). Rapid microwave-assisted synthesis of hybrid zeolitic–imidazolate frameworks with mixed metals and mixed linkers. J. Mater. Chem. A.

[cit29] Bogani L., Vindigni A., Sessoli R., Gatteschi D. (2008). Single chain magnets: where to from here?. J. Mater. Chem..

[cit30] Caneschi A., Gatteschi D., Lalioti N., Sangregorio C., Sessoli R., Venturi G., Vindigni A., Rettori A., Pini M. G., Novak M. A. (2001). Cobalt(II)-Nitronyl Nitroxide Chains as Molecular Magnetic Nanowires. Angew. Chem., Int. Ed..

[cit31] Liu X., Sun L., Zhou H., Cen P., Jin X., Xie G., Chen S., Hu Q. (2015). Single-Ion-Magnet Behavior in a Two-Dimensional Coordination Polymer Constructed from Co II Nodes and a Pyridylhydrazone Derivative. Inorg. Chem..

[cit32] Zhu Y.-Y., Zhu M.-S., Yin T.-T., Meng Y.-S., Wu Z.-Q., Zhang Y.-Q., Gao S. (2015). Cobalt(ii) Coordination Polymer Exhibiting Single-Ion-Magnet-Type Field-Induced Slow Relaxation Behavior. Inorg. Chem..

[cit33] Chahine A. Y., Phonsri W., Murray K. S., Turner D. R., Batten S. R. (2020). Coordination polymers of a bis-isophthalate bridging ligand with single molecule magnet behaviour of the CoII analogue. Dalton Trans..

[cit34] Konieczny P., Gonzalez-Guillén A. B., Luberda-Durnaś K., Čižmár E., Pełka R., Oszajca M., Łasocha W. (2019). 1D coordination polymer (OPD) 2 Co II SO 4 showing SMM behaviour and multiple relaxation modes. Dalton Trans..

[cit35] Gatteschi D. , Sessoli R. and Villain J., Molecular Nanomagnets, Oxford University PressEditor, 2006, 10.1093/acprof:oso/9780198567530.001.0001

[cit36] Atzori M., Sessoli R. (2019). The Second Quantum Revolution: Role and Challenges of Molecular Chemistry. J. Am. Chem. Soc..

[cit37] Coronado E. (2020). Molecular magnetism: from chemical design to spin control in molecules, materials and devices. Nat. Rev. Mater..

[cit38] Juanhuix J., Gil-Ortiz F., Cuní G., Colldelram C., Nicolás J., Lidón J., Boter E., Ruget C., Ferrer S., Benach J. (2014). Developments in optics and performance at BL13-XALOC, the macromolecular crystallography beamline at the Alba Synchrotron. J. Synchrotron Radiat..

[cit39] Kabsch W. (2010). XDS. Acta Crystallogr., Sect. D: Biol. Crystallogr..

[cit40] Sheldrick G. M. (2015). Crystal structure refinement with SHELXL. Acta Crystallogr., Sect. C: Struct. Chem..

[cit41] Dolomanov O. V., Bourhis L. J., Gildea R. J., Howard J. K., Puschmann H. (2009). OLEX2: a complete structure solution, refinement and analysis program. J. Appl. Crystallogr..

[cit42] Galván I. F., Vacher M., Alavi A., Angeli C., Aquilante F., Autschbach J., Bao J. J., Bokarev S. I., Bogdanov N. A., Carlson R. K., Chibotaru L. F., Creutzberg J., Dattani N., Delcey M. G., Dong S. S., Dreuw A., Freitag L., Frutos L. M., Gagliardi L., Gendron F., Giussani A., González L., Grell G., Guo M., Hoyer C. E., Johansson M., Keller S., Knecht S., Kovačević G., Källman E., Li Manni G., Lundberg M., Ma Y., Mai S., Malhado J. P., Malmqvist P. Å., Marquetand P., Mewes S. A., Norell J., Olivucci M., Oppel M., Phung Q. M., Pierloot K., Plasser F., Reiher M., Sand A. M., Schapiro I., Sharma P., Stein C. J., Sørensen L. K., Truhlar D. G., Ugandi M., Ungur L., Valentini A., Vancoillie S., Veryazov V., Weser O., Wesołowski T. A., Widmark P.-O., Wouters S., Zech A., Zobel J. P., Lindh R. (2019). OpenMolcas: From Source Code to Insight. J. Chem. Theory Comput..

[cit43] Neese F. (2012). The ORCA program system. Wiley Interdiscip. Rev.: Comput. Mol. Sci..

[cit44] Malmqvist P. Å., Roos B. O., Schimmelpfennig B. (2002). The restricted active space (RAS) state interaction approach with spin–orbit coupling. Chem. Phys. Lett..

[cit45] Roos B. O., Lindh R., Malmqvist P.-Å., Veryazov V., Widmark P.-O., Borin A. C. (2008). New Relativistic Atomic Natural Orbital Basis Sets for Lanthanide Atoms with Applications to the Ce Diatom and LuF 3. J. Phys. Chem. A.

[cit46] Roos B. O., Lindh R., Malmqvist P. Å., Veryazov V., Widmark P. O. (2004). Main Group Atoms and Dimers Studied with a New Relativistic ANO Basis Set. J. Phys. Chem. A.

[cit47] Widmark P. O., Malmqvist P. Å., Roos B. O. (1990). Density matrix averaged atomic natural orbital (ANO) basis sets for correlated molecular wave functions. Theor. Chim. Acta.

[cit48] Ungur L., Chibotaru L. F. (2016). Strategies toward High-Temperature Lanthanide-Based Single-Molecule Magnets. Inorg. Chem..

[cit49] Schaefer A., Huber C., Ahlrichs R. (1994). Fully optimized contracted Gaussian basis sets of triple zeta valence quality for atoms Li to Kr. J. Chem. Phys..

[cit50] Weigend F. (2006). Accurate Coulomb-fitting basis sets for H to Rn. Phys. Chem. Chem. Phys..

[cit51] Cordero B., Gómez V., Platero-Prats A. E., Revés M., Echeverría J., Cremades E., Barragán F., Alvarez S. (2008). Covalent radii revisited. Dalton Trans..

[cit52] Howe J. D., Morelock C. R., Jiao Y., Chapman K. W., Walton K. S., Sholl D. S. (2017). Understanding Structure, Metal Distribution, and Water Adsorption in Mixed-Metal MOF-74. J. Phys. Chem. C.

[cit53] Cano A., Lartundo-Rojas L., Shchukarev A., Reguera E. (2019). Contribution to the coordination chemistry of transition metal nitroprussides: a cryo-XPS study. New J. Chem..

[cit54] Wang M., Gou X., Shi W., Cheng P. (2019). Single-chain magnets assembled in cobalt(ii) metal–organic frameworks. Chem. Commun..

[cit55] Sieklucka B. , Pinkowicz D., Pedersen K. S., Vindigni A., Sessoli R., Coulon C. and Clérac R., Molecular Magnetic Materials Molecular Multiferroics, ed. B. Sieklucka and Pinkowicz D., 2016, ch. 6, 10.1002/9783527694228

[cit56] Pedersen K. S., Bendix J., Clérac R. (2014). Single-molecule magnet engineering:
building-block approaches. Chem. Commun..

[cit57] Madhu N. T., Tang J.-K., Hewitt I. J., Clérac R., Wernsdorfer W., Van Slageren J., Anson C. E., Powell A. K. (2005). What makes a single molecule magnet?. Polyhedron.

[cit58] Chen L., Song J., Zhao W., Yi G., Zhou Z., Yuan A., Song Y., Wang Z., Ouyang Z.-W. (2018). A mononuclear five-coordinate Co(ii) single molecule magnet with a spin crossover between the *S* = 1/2 and 3/2 states. Dalton Trans..

[cit59] El-Khatib F., Cahier B., Shao F., López-Jordà M., Guillot R., Rivière E., Hafez H., Saad Z., Girerd J.-J., Guihéry N., Mallah T. (2017). Design and Magnetic Properties of a Mononuclear Co(ii) Single Molecule Magnet and Its Antiferromagnetically Coupled Binuclear Derivative. Inorg. Chem..

[cit60] Díaz-Torres R., Menelaou M., Roubeau O., Sorrenti A., Brandariz-de-Pedro G., Sañudo E. C., Teat S. J., Fraxedas J., Ruiz E., Aliaga-Alcalde N. (2016). Multiscale study of mononuclear Co II SMMs based on curcuminoid ligands. Chem. Sci..

[cit61] Saber M. R., Dunbar K. R. (2014). Ligands effects on the magnetic anisotropy of tetrahedral cobalt complexes. Chem. Commun..

[cit62] Deng Y.-F., Singh M. K., Gan D., Xiao T., Wang Y., Liu S., Wang Z., Ouyang Z., Zhang Y.-Z., Dunbar K. R. (2020). Probing the Axial Distortion Effect on the Magnetic Anisotropy of Octahedral Co(ii) Complexes. Inorg. Chem..

[cit63] Rigamonti L., Bridonneau N., Poneti G., Tesi L., Sorace L., Pinkowicz D., Jover J., Ruiz E., Sessoli R., Cornia A. (2018). A Pseudo-Octahedral Cobalt(II) Complex with Bispyrazolylpyridine Ligands Acting as a Zero-Field Single-Molecule Magnet with Easy Axis Anisotropy. Chem.–Eur. J..

[cit64] Váhovská L., Vitushkina S., Potočňák I., Trávníček Z., Herchel R. (2018). Effect of linear and non-linear pseudohalides on the structural and magnetic properties of Co(ii) hexacoordinate single-molecule magnets. Dalton Trans..

[cit65] Wei H.-W., Yang Q.-F., Lai X.-Y., Wang X.-Z., Yang T.-L., Hou Q., Liu X.-Y. (2018). Field-induced slow relaxation of magnetization in a distorted octahedral mononuclear high-spin Co(ii) complex. CrystEngComm.

[cit66] Gomez-Coca S., Cremades E., Aliaga-Alcalde N., Ruiz E. (2013). Mononuclear Single-Molecule Magnets: Tailoring the Magnetic Anisotropy of First-Row Transition-Metal Complexes. J. Am. Chem. Soc..

[cit67] Roy S., Oyarzabal I., Vallejo J., Cano J., Colacio E., Bauza A., Frontera A., Kirillov A. M., Drew M. G. B., Das S. (2016). Two Polymorphic Forms of a Six-Coordinate Mononuclear Cobalt(ii) Complex with Easy-Plane Anisotropy: Structural Features, Theoretical Calculations, and Field-Induced Slow Relaxation of the Magnetization. Inorg. Chem..

[cit68] Palacios M. A., Nehrkorn J., Suturina E. A., Ruiz E., Gómez-Coca S., Holldack K., Schnegg A., Krzystek J., Moreno J. M., Colacio E. (2017). Analysis of Magnetic Anisotropy and the Role of Magnetic Dilution in Triggering Single-Molecule Magnet (SMM) Behavior in a Family of Co II Y III Dinuclear Complexes with Easy-Plane Anisotropy. Chem.–Eur. J..

[cit69] Lloret F., Julve M., Cano J., Ruiz-García R., Pardo E. (2008). Magnetic properties of six-coordinated high-spin cobalt(II) complexes: Theoretical background and its application. Inorg. Chim. Acta.

[cit70] Masegosa A., Palacios M. A., Ruiz E., Gómez-Coca S., Krzystek J., Moreno J. M., Colacio E. (2019). Dinuclear Co II Y III *vs.* tetranuclear CoII2YIII2 complexes: the effect of increasing molecular size on magnetic anisotropy and relaxation dynamics. Dalton Trans..

[cit71] Vallejo J., Castro I., Ruiz-García R., Cano J., Julve M., Lloret F., De Munno G., Wernsdorfer W., Pardo E. (2012). Field-Induced Slow Magnetic Relaxation in a Six-Coordinate Mononuclear Cobalt(ii) Complex with a Positive Anisotropy. J. Am. Chem. Soc..

[cit72] Chilton N. F., Anderson R. P., Turner L. D., Soncini A., Murray K. S. (2013). PHI: A powerful new program for the analysis of anisotropic monomeric and exchange-coupled polynuclear d - and f -block complexes. J. Comput. Chem..

[cit73] Wang J.-Y., Shi Y., Tao D.-L., Yin G.-Y., Bo Q.-B. (2020). 2D chain layer *versus* 1D chain: rigid aromatic benzoate disassembling flexible alicyclic dicarboxylate-based lanthanide coordination polymers with enhanced photoluminescence and characteristic single-molecule magnet behavior. CrystEngComm.

[cit74] Orts-Arroyo M., Castro I., Lloret F., Martínez-Lillo J. (2020). Field-induced slow relaxation of magnetisation in two one-dimensional homometallic dysprosium(iii) complexes based on alpha- and beta-amino acids. Dalton Trans..

[cit75] Cornia A., Barra A.-L., Bulicanu V., Clérac R., Cortijo M., Hillard E. A., Galavotti R., Lunghi A., Nicolini A., Rouzières M., Sorace L., Totti F. (2020). The Origin of Magnetic Anisotropy and Single-Molecule Magnet Behavior in Chromium(ii)-Based Extended Metal Atom Chains. Inorg. Chem..

[cit76] Jassal A. K., Aliaga-Alcalde N., Corbella M., Aravena D., Ruiz E., Hundal G. (2015). Neodymium 1D systems: targeting new sources for field-induced slow magnetization relaxation. Dalton Trans..

[cit77] Mondal P., Dey B., Roy S., Bera S. P., Nasani R., Santra A., Konar S. (2018). Field-Induced Slow Magnetic Relaxation and Anion/Solvent Dependent Proton Conduction in Cobalt(ii) Coordination Polymers. Cryst. Growth Des..

[cit78] Díaz-Torres R., Menelaou M., González-Campo A., Teat S., Sañudo E., Soler M., Aliaga-Alcalde N. (2016). Comparative Magnetic Studies in the Solid State and Solution of Two Isostructural 1D Coordination Polymers Containing CoII/NiII-Curcuminoid Moieties. Magnetochemistry.

[cit79] Wu Y., Tian D., Ferrando-Soria J., Cano J., Yin L., Ouyang Z., Wang Z., Luo S., Liu X., Pardo E. (2019). Modulation of the magnetic anisotropy of octahedral cobalt single-ion magnets by fine-tuning the axial coordination microenvironment. Inorg. Chem. Front..

[cit80] Palion-Gazda J., Choroba K., Machura B., Świtlicka A., Kruszynski R., Cano J., Lloret F., Julve M. (2019). Influence of the pyrazine substituent on the structure and magnetic properties of dicyanamide-bridged cobalt(ii) complexes. Dalton Trans..

[cit81] Świtlicka A., Palion-Gazda J., Machura B., Cano J., Lloret F., Julve M. (2019). Field-induced slow magnetic relaxation in pseudooctahedral cobalt(ii) complexes with positive axial and large rhombic anisotropy. Dalton Trans..

[cit82] Vallejo J., Viciano-Chumillas M., Lloret F., Julve M., Castro I., Krzystek J., Ozerov M., Armentano D., De Munno G., Cano J. (2019). Coligand Effects on the Field-Induced Double Slow Magnetic Relaxation in Six-Coordinate Cobalt(ii) Single-Ion Magnets (SIMs) with Positive Magnetic Anisotropy. Inorg. Chem..

[cit83] Aravena D., Ruiz E. (2020). Spin dynamics in single-molecule magnets and molecular qubits. Dalton Trans..

[cit84] Scott P. L., Jeffries C. D. (1962). Spin-Lattice Relaxation in Some Rare-Earth Salts at Helium Temperatures; Observation of the Phonon Bottleneck. Phys. Rev..

[cit85] Gómez-Coca S., Aravena D., Morales R., Ruiz E. (2015). Large magnetic anisotropy in mononuclear metal complexes. Coord. Chem. Rev..

