# Supplementary material for: Single molecule magnets of cobalt and zinc homo- and heterometallic coordination polymers prepared by a one-step synthetic procedure
Source: RSC Adv. 2020 Dec 21;10(73):45090–104. doi: 10.1039/d0ra09132d (PMC9058601; doi:10.1039/d0ra09132d)
Supplement: RA-010-D0RA09132D-s001 [file RA-010-D0RA09132D-s001.pdf]

Electronic Supplementary Material (ESI) for RSC Advances.
This journal is © The Royal Society of Chemistry 2020

Single molecule magnets of cobalt and zinc homo- and heterometallic coordination polymers prepared by one-step synthetic procedure

Núria Portolés-Gil,[a] Silvia Gómez-Coca,[b] Oriol Vallcorba,[c] Gregorio Marbán,[d] Núria Aliaga-Alcalde,[a,e] Ana López-Periago,[a] José A. Ayllón,[f*] and Concepción Domingo[a,*]

[a] *Instituto de Ciencia de Materiales de Barcelona (CSIC), Campus UAB, 08193 Bellaterra, Spain.*

[b] *Departament de Química Inorgànica i Orgànica and Institut de Recerca de Química Teòrica i Computacional, Universitat de Barcelona, Diagonal 645, 08028 Barcelona, Spain*

[c] *ALBA Synchrotron Light Source, 08290 Cerdanyola del Vallés, Spain.*

[d] *Instituto de Ciencia y Tecnología del Carbono (INCAR-CSIC), 33011 Oviedo, Spain;*

[e] *ICREA, Institució Catalana de Recerca i Estudis Avançats, Passeig Lluis Companys 23, 08010 Barcelona, Spain.*

[f] *Universidad Autónoma de Barcelona, Dept. Química, Campus UAB, 08193 Bellaterra, Spain.*

Table S1. Crystallographic S-SXRD data for **Zn(1)**, **Zn/Co(4)** and **Co(2)**.

	**Zn(1)**	**Zn/Co(4)**	**Co(2)**
Molecular formula (MF)	$C_{28}H_{18}F_{12}N_2O_4Zn$	$C_{28}H_{18}F_{12}N_2O_4Co_{0.47}Zn_{0.53}$	$C_{28}H_{18}F_{12}N_2O_4Co$
Formula weight	739.82	736.82	733.38
Crystal system, space group	Triclinic, P $\bar{1}$	Triclinic, P $\bar{1}$	Triclinic, P $\bar{1}$
a (Å)	8.900(2)	8.930(7)	8.77040(7)
b (Å)	9.8940(13)	9.880(5)	9.15990(6)
c (Å)	17.5110(17)	17.570(9)	10.04300(11)
α (º)	104.699(10)	104.57(6)	62.9720(6)
β (º)	96.02(2)	96.38(3)	71.4100(7)
γ (º)	97.45(3)	97.45(8)	80.8890(6)
V (Å^3)	1463.6(5)	1470.9(17)	681.113(11)
Z (according to MF)	2	2	1
D_{calc} (g·cm^{-3})	1.679	1.664	1.788
T (K)	100	100	100
Wavelength (Å)	0.72932	0.72929	0.72931
μ (mm^{-1})	1.016	0.869	0.799
F(000)	740	737	367
Crystal size (mm^3)	0.13x0.06x0.04	0.13x0.08x0.06	0.10x0.06x0.03
hkl ranges	-12≤h≤12<br>-14≤k≤14<br>-24≤l≤24	-12≤h≤12<br>-13≤k≤13<br>-24≤l≤24	-13≤h≤13<br>-14≤k≤14<br>-14≤l≤14
θ range (º)	2.213 to 31.393	1.243 to 30.427	2.515 to 34.125
Reflections collected/ unique [R_{int}]	39373/8283 [0.056]	104899/8256 [0.053]	40318/4595 [0.036]
Completeness to θ (%)	95.4	99.8	99.6
Data/restrains/parameters	8283/31/438	8256/0/434	4595/0/215
Goodness of fit on F^2	1.063	1.065	1.096
R_1 / wR_2 [I>2σ(I)]	0.0331/0.0838	0.0348/0.0964	0.0288/0.0790
R_1 / wR_2 (*all data*)	0.0348/0.0850	0.0357/0.0971	0.0289/0.0794
Largest. Diff. peak and hole (e Å^{-3})	0.657 and -0.496	0.796 and -0.600	0.549 and -0.607

Table S2. Crystallographic S-PXRD data **Co(3)**.

	**Co(3)**
Molecular formula (MF)	$C_{28}H_{18}F_{12}N_2O_4Co$
Formula weight	733.38
Crystal system, space group	Triclinic, P $\bar{1}$
a (Å)	8.9127(4)
b (Å)	9.8610(5)
c (Å)	17.5409(9)
α (º)	104.4(2)
β (º)	96.3(2)
γ (º)	97.5(2)
V (Å^3)	1464.1(19)
Z (according to MF)	2
D_{calc} (g·cm^{-3})	1.664
T (K)	100
Wavelength (Å)	0.61978
μ (mm^{-1})	0.482
F(000)	734.0
Meas. 2θ range, stepsize (º)	1.026 to 45.246, 0.006
Profile function	Pseudo-Voigt
2θ range used (º)	1.800 to 34.998
Num. of reflections	2803
Data points	5533
Restrains/parameters	172/156
R_{wp}	0.077
R_{exp}	0.010
$\chi_{Rietveld}$ / $\chi_{Pattern\_matching}$	1.90

Table S3. Cartesian coordinates of the molecular models used to study de magnetic properties of compound *cis*-[Co(hfacac)$_2$bpymb]$_n$, **Co(3).**

Co	0.00000	0.00000	0.00000
O	0.00000	0.00000	1.96547
O	-0.14944	-2.05491	0.09459
C	-0.72996	-0.41952	4.20129
C	-0.51976	-0.83947	2.73984
C	-0.95361	-2.10315	2.32769
H	-1.39240	-2.64633	2.94228
C	-0.77140	-2.60442	1.03356
C	-1.34766	-3.99928	0.73342
F	0.25772	0.33420	4.64679
F	-1.82719	0.33367	4.31540
F	-0.91140	-1.41988	5.04795
F	-2.36977	-4.33948	1.51356
F	-1.79571	-4.07272	-0.51200
F	-0.40951	-4.92899	0.83835
O	-2.06615	0.10173	-0.21273
O	0.09674	-0.22929	-2.13727
C	-4.18621	-0.73065	-0.74025
C	-2.71070	-0.49073	-1.11679
C	-2.18533	-0.90622	-2.35681
H	-2.77130	-1.31919	-2.95018
C	-0.85445	-0.74582	-2.77960
C	-0.44504	-1.24712	-4.17910
F	-4.87853	-1.30530	-1.70814
F	-4.30732	-1.53310	0.32136
F	-4.79184	0.41096	-0.43021
F	-1.49035	-1.71530	-4.86648
F	0.44197	-2.24512	-4.07279
F	0.10574	-0.28335	-4.91431
N	-0.00361	2.16078	-0.06876
C	0.58017	2.90016	-1.03170
H	0.97759	2.46222	-1.74943
C	0.61101	4.28567	-0.98805
H	1.02840	4.75164	-1.67594
C	0.03070	4.98820	0.07023
C	-0.57332	4.22224	1.05895
H	-0.97821	4.63671	1.78688
C	-0.57012	2.83911	0.95057
H	-0.98588	2.35285	1.62515
C	0.05856	6.51051	0.13312
H	0.83098	6.84068	-0.35401
H	-0.74586	6.87680	-0.26543
H	0.11912	6.77987	1.06372
N	2.15893	-0.10975	-0.22591

C	2.88383	-1.18855	-0.56316
H	2.42428	-1.93042	-0.88477
C	4.26642	-1.27117	-0.46404
H	4.71380	-2.04826	-0.71159
C	4.96521	-0.16517	0.00826
C	4.23293	0.96374	0.35949
H	4.66767	1.72056	0.68084
C	2.85570	0.94743	0.22653
H	2.38626	1.71333	0.46717
C	6.47381	-0.13762	0.15862
H	6.71284	-0.46127	1.04043
H	6.87013	-0.71813	-0.51082
H	6.81950	0.76160	0.03981

Table S4. Cartesian coordinates of the molecular models used study de magnetic properties of compound *trans*-[Co(hfacac)$_2$bpymb]$_n$, **Co(2).**

Co	0.00000	0.00000	0.00000
F	1.74075	-5.06176	-0.48644
F	-0.18462	-4.54014	-1.29977
F	0.08343	-4.68385	0.83347
F	4.88077	-1.76214	-1.21426
F	4.31026	0.29017	-1.49318
O	-0.00004	-2.06198	-0.00378
O	2.06381	-0.05124	-0.03974
F	4.86735	-0.40677	0.45099
N	-0.00000	0.00000	-2.15520
C	0.99731	-2.78055	-0.24540
C	2.73505	-1.06497	-0.36921
C	2.32320	-2.39475	-0.48718
H	2.94526	-3.04080	-0.73202
C	1.02472	0.52530	-2.85274
H	1.72838	0.90942	-2.38099
C	-1.03779	-0.55424	-4.25591
H	-1.76656	-0.91793	-4.70446
C	0.03908	-0.03292	-4.97715
C	1.08393	0.52250	-4.23932
H	1.81880	0.89002	-4.67490
C	4.21279	-0.74771	-0.66171
C	-1.01798	-0.52840	-2.87129
H	-1.73994	-0.89117	-2.41089
H	0.91601	-0.21223	-6.88494
C	0.66786	-4.28579	-0.29299
C	0.04243	-0.05794	-6.49258
H	-0.30031	0.78892	-6.81821
H	-0.56093	-0.75427	-6.79365
F	-1.74075	5.06176	0.48644

F	0.18462	4.54014	1.29977
F	-0.08343	4.68385	-0.83347
F	-4.88076	1.76214	1.21426
F	-4.31026	-0.29017	1.49318
O	0.00004	2.06198	0.00379
O	-2.06381	0.05124	0.03974
F	-4.86735	0.40677	-0.45099
C	-0.99731	2.78055	0.24540
C	-2.73505	1.06497	0.36921
C	-2.32320	2.39475	0.48718
H	-2.94526	3.04080	0.73202
C	-4.21279	0.74771	0.66171
C	-0.66786	4.28579	0.29299
N	0.00000	-0.00000	2.15520
C	-1.02472	-0.52530	2.85274
H	-1.72838	-0.90942	2.38099
C	1.03779	0.55424	4.25591
H	1.76656	0.91793	4.70446
C	-0.03908	0.03292	4.97715
C	-1.08393	-0.52250	4.23932
H	-1.81880	-0.89002	4.67490
C	1.01798	0.52840	2.87129
H	1.73994	0.89117	2.41089
H	-0.91601	0.21223	6.88494
C	-0.04243	0.05794	6.49258
H	0.30031	-0.78892	6.81821
H	0.56093	0.75427	6.79365

Table S5. Cartesian coordinates of the molecular models used study de magnetic properties of compound *trans*- *cis*-[$Zn_{0.5}Co_{0.5}(hfacac)_2$bpymb], **Zn/Co(4).**

Co	0.00000	0.00000	0.00000
O	0.00000	0.00000	1.91627
O	-0.22028	-2.07073	0.12049
C	-0.75087	-0.37653	4.15167
C	-0.54007	-0.81251	2.70029
C	-0.99604	-2.08566	2.35565
H	-1.42929	-2.61467	2.98721
C	-0.82157	-2.60290	1.07409
C	-1.36880	-3.98370	0.70403
F	0.28133	0.29192	4.62870
F	-1.79285	0.45405	4.25444
F	-1.03818	-1.36280	4.98440
F	-2.37969	-4.38192	1.46885
F	-1.82485	-4.00372	-0.53801
F	-0.42606	-4.91149	0.78157

O	-2.10490	0.08828	-0.25027
O	0.05599	-0.22782	-2.20691
C	-4.20959	-0.84576	-0.72397
C	-2.74957	-0.55219	-1.12902
C	-2.24465	-0.96576	-2.38293
H	-2.81696	-1.40647	-2.96889
C	-0.91788	-0.74468	-2.79942
C	-0.50231	-1.22641	-4.20622
F	-4.94101	-1.38532	-1.68281
F	-4.26756	-1.66480	0.33069
F	-4.81196	0.27959	-0.35484
F	-1.56636	-1.63102	-4.90865
F	0.33671	-2.26310	-4.09543
F	0.10311	-0.26827	-4.90238
N	-0.00868	2.12724	-0.13570
C	0.48085	2.87161	-1.14197
H	0.83230	2.43559	-1.88507
C	0.50058	4.25581	-1.11343
H	0.85363	4.74247	-1.82322
C	-0.01433	4.90103	0.01606
C	-0.52456	4.12112	1.04267
H	-0.88296	4.53175	1.79661
C	-0.51688	2.74208	0.94819
H	-0.87570	2.23379	1.63949
C	-0.00637	6.42680	0.08893
H	0.72608	6.75996	-0.45396
H	-0.84158	6.77524	-0.25776
H	0.11175	6.72489	1.00441
N	2.14700	-0.12629	-0.28035
C	2.91960	-1.17231	-0.61441
H	2.46817	-1.91928	-0.93777
C	4.30043	-1.26943	-0.53330
H	4.73598	-2.05123	-0.78399
C	5.02350	-0.17542	-0.07422
C	4.25110	0.92682	0.27273
H	4.68002	1.68844	0.59118
C	2.87455	0.91648	0.16021
H	2.41473	1.68745	0.40670
C	6.53561	-0.17373	0.04495
H	6.78914	-0.49305	0.92522
H	6.92453	-0.75672	-0.62674
H	6.86693	0.72874	-0.08414

XPS results and discussion

Atomic surface compositions for Zn(sc), Co(Et) and Zn/Co(sc) samples were measured by XPS. Results are shown in Table S6. Spectra for the Zn2p (1015-1055 eV), Co2p (775-810 eV) and C1s (280-300 eV) regions were deconvoluted for analysis (Fig. S2), together with the O1s spectrum (525-540 eV, not shown). The results of the deconvolution procedure for the C1s region were performed on the basis of following peak assignations: 284.6 eV (C=C (sp^2 bonds, reference peak),[1] 285.6 eV (C-H sp^3 bonds and/or carbon atoms in C-N functional groups),[2,3] 287.4 eV (C-O bonds),[4] and 292.4 eV (CF_3 bonds in hfacac-based compounds).[5]

Zn(sc) sample: the C/N, O/N and F/N ratios on the surface were almost coincident with the ratios calculated for the structural unit [Zn(hfacac)$_2$bpymb]. However, the measured value of the C/Zn ratio was much lower than the theoretical one. This suggests an excess of zinc on the surface. The binding energy of 1021.9 eV read at the maximum of the peak in the Zn2p3/2 region is usually ascribed to Zn-O bond.[6] Furthermore, the difference in binding energy between the maximum of the Zn2p1/2 region (1045.0 eV) and that of the Zn2p3/2 region is 23.1 eV, typical of divalent Zn.[7] Finally, the Auger parameter evaluated from the ZnLMM spectrum (2008.9 eV) clearly corroborates the divalent state of Zn. Fig. S2a shows small peaks in the Zn2p3/2 and Zn2p1/2 regions just to the right of the peaks associated to Zn-O (1023.4 and 1046.5 eV, respectively). The peak at 1023.4 eV is associated to $Zn(OH)_2$,[8] and must be a product of the degradation process. In the O1s spectrum, oxygen in $Zn(OH)_2$ represents about 33% of the area of the peak at 531.7 eV,[9] the remainder being ascribed to the >C=O bonds.[10] The presence of $Zn(OH)_2$ was not detected in the original sample by techniques such as XRD or E.A. Hence, its formation can only be explained by the occurrence of a significant degradation of the surface of the sample during X-ray irradiation. The degradation percentage on the surface during XPS measurement was calculated by mass balance of the total Zn(II), O, C, F and N content assuming that the irradiated surface was composed of [Zn(hfacac)$_2$bpymb]

(C1), $Zn(OH)_2$ (C2) and a generic compound $Zn_{q0}C_{q1}F_{q2}O_{q3}N_{q4}$ (C3). For the speciation of zinc (Zn in C1, C2 and C3), it was assumed that the ratio of the area of peaks at 1021.9 eV and 1023.4 eV was equal to ([C1]+q_0×[C3])/[C2]. Added to this, the percentage of carbon associated to the peak at 287.4 eV in the C1s region (C-O bonds) must be equal to 4×[C1]+m×q_3×[C3], where m is a variable to be optimized that represents the O/C ratio in the C-O bonds existing in C3. In these calculations, q_3 resulted to be 0, so that the value of m became irrelevant. The weight loss during the degradation process was around 35 wt.%. The combination of all the species released in the process has the average formula of $C_{14}F_6O_2N$, which might correspond to the breakage of bpymb linker and the liberation of hafacac ligands. The degradation process during the analysis transformed around 52 mol% of the original $Zn(hafacac)_2bpymb$ into $Zn(OH)_2$ (~24 mol%) and $ZnC_{14}F_6N$ (~28 mol%).

Co(Et) sample: the C/N and O/N ratios on the surface were only a bit over the expected ratios for the structural unit [$Co(hafacac)_2bpymb$]. However, the F/N ratio was much larger than the theoretical one (8.5 *vs*. 6) and the C/Co ratio much lower (21.6 *vs*. 28). In this case, the cobalt speciation in the Co2p region could not be performed due to the lack of clear specific peaks in the spectrum representing the different species. Taking into account that Co(II) in the Co2p3/3 and Co2p1/2 displayed a bonding environment almost identical to that of $Co(OH)_2$,[11] it is reasonable to assume that a significant percentage of the polymer on the surface was again degraded by XPS irradiation. The degradation was also responsible for the non-stoichiometry in the C/Co and F/N ratios. Assuming that the Co(Et) degraded sample is composed of [$Co(hafacac)_2bpymb$] (C1), $Co(OH)_2$ (C2) and a generic compound $Co_{q0}C_{q1}F_{q2}O_{q3}N_{q4}$ (C3), a mass balance for the total Co(II), O, C, F and N contents allows to evaluate the values of q_0 to q_4, as well as the molar concentrations of C1, C2, and C3. The percentage of C associated to the peak at 287.4 eV in the C1s region (C-O bonds) must be equal to 4×[C1]+m×q_3×[C3], where m is a variable to be optimized that represents the O/C ratio in the C-O bonds existing in C3. In

these calculations, the minimum error was achieved with m=1. In the degraded sample the Co(II) was present only in C1 and C2, since q_0=0. The weight loss during the degradation process was around 11 wt.%. The combination of all the species released in the process has the average formula of $C_{13}O_{1.3}N$. Around 57 mol% of the original [Co(hafacac)$_2$bpymb] was converted to $Co(OH)_2$ during the degradation process.

Zn/Co(sc) sample: the C/N and O/N ratios were much higher than those expected for the structural unit [$Zn_{0.5}Co_{0.5}$(hafacac)$_2$bpymb]. Furthermore, the F/N ratio was also much higher than 6 and the C/(Co+Zn) ratio was well below 28. All these facts indicated that this product also suffers from degradation during the analysis. The shapes, position and areas of the different XPS peaks in the Zn2p and Co2p regions pointed to decomposition products similar to those detected for the homometallic compounds. Under this assumption, the balance for the total Co(II), Zn(II), C, O, F and N indicates that the original [Co(hafacac)$_2$bpymb] was converted to $Co(OH)_2$ during the degradation process in *ca.* 82 mol%, while only *ca.* 40 mol% of the original [Zn(hafacac)$_2$bpymb] decomposed to other Zn-based products. This result is probably related to the higher concentration of Co(II) *vs.* Zn(II) found on the surface, thus leaving this metal favourably exposed for degradation.

Table S6. XPS atomic surface composition of the different studied samples.

	**Zn(sc)**	**Co(Et)**	**Zn/Co(sc)**
Metal	Zn 3.4	Co 2.5	Zn 1.3
			Co 2.1
C	59.1	53.5	56.2
0	8.3	8.4	10.4
N	4.2	3.7	3.0
F	25.0	31.9	26.9
C/N	14.1	14.3	18.6
O/N	2.0	2.2	3.5
C/Metal	17.5	21.6	16.4
F/N	5.9	8.5	8.9

Table S7. Results from the fit of the Cole-Cole plot, for compound **Co(2)** with an applied dc field of 1500 Oe, to a Debye model using the CCfit package.

Temperature	Xs	Xt	tau	alpha	residual
1.8	3.46E-02	9.46E-01	6.43E-04	6.85E-02	9.85E-04
1.9	3.22E-02	9.07E-01	5.90E-04	7.31E-02	7.60E-04
2	3.21E-02	8.63E-01	5.35E-04	7.31E-02	6.80E-04
2.1	3.12E-02	8.23E-01	4.87E-04	7.40E-02	7.58E-04
2.3	3.06E-02	7.61E-01	4.14E-04	7.51E-02	7.38E-04
2.5	3.07E-02	7.03E-01	3.50E-04	7.78E-02	5.77E-04
2.7	3.08E-02	6.53E-01	2.98E-04	7.85E-02	5.23E-04
2.9	3.24E-02	6.10E-01	2.56E-04	7.77E-02	4.42E-04
3.1	3.33E-02	5.72E-01	2.21E-04	7.79E-02	4.15E-04
3.3	3.55E-02	5.39E-01	1.91E-04	7.84E-02	3.21E-04
3.5	3.92E-02	5.10E-01	1.67E-04	7.60E-02	2.94E-04
3.7	4.35E-02	4.83E-01	1.46E-04	7.39E-02	2.18E-04
4	4.70E-02	4.48E-01	1.20E-04	7.44E-02	1.67E-04
4.31	5.71E-02	4.19E-01	1.01E-04	6.82E-02	1.18E-04
4.6	6.86E-02	3.93E-01	8.68E-05	6.12E-02	8.68E-05
5	8.59E-02	3.62E-01	7.27E-05	4.85E-02	3.80E-05
5.5	9.45E-02	3.31E-01	5.68E-05	3.95E-02	1.13E-05
6	1.09E-01	3.04E-01	4.67E-05	2.50E-02	4.81E-06
6.5	1.19E-01	2.82E-01	3.80E-05	1.94E-02	4.61E-06
7	1.33E-01	2.62E-01	3.20E-05	2.25E-02	2.59E-06
8	1.34E-01	2.31E-01	1.79E-05	7.74E-02	6.28E-06

Table S8. Results from the fit of the Cole-Cole plot, for compound **Co(3)** with an applied dc field of 1500 Oe, to a Debye model using the CCfit package.

Temperature	Xs	Xt	tau	alpha	residual
1.8	3.64E-02	9.54E-01	7.36E-04	5.04E-02	7.73E-04
1.9	3.54E-02	9.14E-01	6.79E-04	5.26E-02	8.20E-04
2	3.51E-02	8.70E-01	6.17E-04	5.28E-02	6.80E-04
2.1	3.46E-02	8.30E-01	5.62E-04	5.43E-02	7.08E-04
2.3	3.47E-02	7.67E-01	4.78E-04	5.50E-02	6.20E-04
2.5	3.45E-02	7.08E-01	4.02E-04	5.58E-02	5.10E-04
2.7	3.54E-02	6.58E-01	3.40E-04	5.49E-02	3.88E-04
2.9	3.65E-02	6.15E-01	2.88E-04	5.21E-02	2.97E-04
3.1	3.72E-02	5.77E-01	2.44E-04	5.00E-02	2.34E-04
3.3	3.96E-02	5.43E-01	2.07E-04	4.53E-02	1.64E-04
3.5	4.03E-02	5.13E-01	1.75E-04	4.17E-02	1.03E-04
3.7	4.35E-02	4.86E-01	1.49E-04	3.70E-02	6.37E-05
4	4.38E-02	4.51E-01	1.15E-04	3.53E-02	3.58E-05
4.3	4.90E-02	4.21E-01	9.01E-05	3.09E-02	2.01E-05
4.6	5.27E-02	3.95E-01	6.99E-05	3.64E-02	2.70E-05
5	7.39E-02	3.64E-01	5.46E-05	2.87E-02	7.37E-06
5.5	9.17E-02	3.33E-01	4.01E-05	2.84E-02	4.90E-06
6	1.26E-01	3.06E-01	3.43E-05	1.50E-02	4.86E-06
6.5	1.41E-01	2.84E-01	2.69E-05	2.85E-02	3.24E-06
7	1.78E-01	2.64E-01	3.01E-05	1.58E-02	3.58E-06
8	2.04E-01	2.33E-01	3.98E-05	1.79E-02	4.27E-06
9	2.00E-01	2.08E-01	7.28E-05	2.05E-02	6.12E-06

Table S9. Results from the fit of the Cole-Cole plot, for compound **Zn/Co(4)** with an applied dc field of 1500 Oe, to a Debye model using the CCfit package.

Temperature	Xs	Xt	tau	alpha	residual
1.8	4.33E-02	5.41E-01	1.31E-03	1.70E-01	1.27E-03
1.9	4.44E-02	5.15E-01	1.17E-03	1.67E-01	9.07E-04
2	4.20E-02	4.90E-01	1.04E-03	1.68E-01	8.46E-04
2.1	4.12E-02	4.68E-01	9.28E-04	1.67E-01	7.37E-04
2.3	4.04E-02	4.32E-01	7.59E-04	1.64E-01	6.86E-04
2.5	4.07E-02	3.98E-01	6.12E-04	1.55E-01	5.46E-04
2.7	4.02E-02	3.70E-01	4.98E-04	1.51E-01	5.71E-04
3	4.26E-02	3.34E-01	3.68E-04	1.30E-01	2.87E-04
3.3	4.39E-02	3.04E-01	2.72E-04	1.13E-01	2.01E-04
3.6	4.47E-02	2.80E-01	2.02E-04	9.71E-02	1.07E-04
3.9	4.78E-02	2.58E-01	1.52E-04	7.58E-02	5.61E-04
4.2	4.74E-02	2.41E-01	1.14E-04	7.58E-02	2.28E-05
4.5	5.02E-02	2.26E-01	8.76E-05	7.58E-02	2.12E-05
5	5.92E-02	2.04E-01	6.10E-05	6.58E-02	1.35E-05
5.5	7.31E-02	1.86E-01	4.85E-05	4.70E-02	5.68E-06
6	8.28E-02	1.71E-01	3.98E-05	3.25E-02	4.39E-06
6.5	9.50E-02	1.59E-01	3.58E-05	2.63E-02	6.28E-06
7	1.06E-01	1.48E-01	3.70E-05	4.20E-14	2.99E-04
8	1.15E-01	1.30E-01	4.59E-05	5.27E-14	4.90E-06

Table S10. Calculated first excitation energies with (δE_1) and without (ΔE_1) spin-orbit coupling (cm^{-1}) and ZFS parameters (D and E in cm^{-1}) and g-factors of the ground and first excited states for the studied molecules at CASSCF level with OpenMolcas and Orca software.

		**OpenMolcas**			**Orca**		
		**2**	**3**	**4**	**2**	**3**	**4**
**ΔE_1 (cm^{-1})**		324	455	392	292	441	382
**δE_1 (cm^{-1})**		231	193	208	239	197	212
**D (cm^{-1})**		114.7	91.2	99.1	118.7	94.3	99.6
**|E| (cm^{-1})**		8.6	18.2	18.5	7.2	17.0	21.1
**KD_1**	**g_x**	5.47	2.43	2.39	3.07	2.39	2.34
	**g_y**	4.50	3.51	3.20	4.29	3.39	3.08
	**g_z**	3.05	6.68	7.00	5.67	6.81	7.13
**KD_2**	**g_x**	0.35	1.23	1.35	0.46	1.32	1.44
	**g_y**	0.39	1.46	1.80	0.57	1.57	1.91
	**g_z**	5.39	5.49	5.21	5.26	5.41	5.13

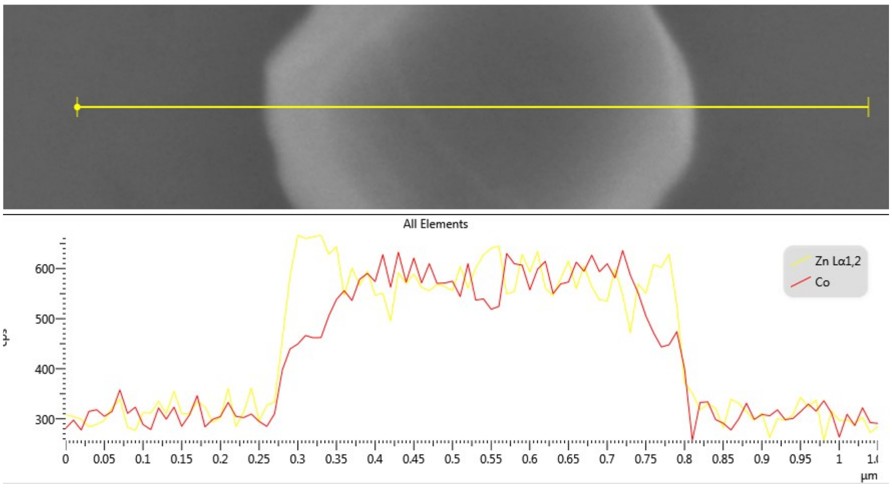

Figure S1. Line scan performed by EDS in a single crystal of the Zn/Co(Et) sample.

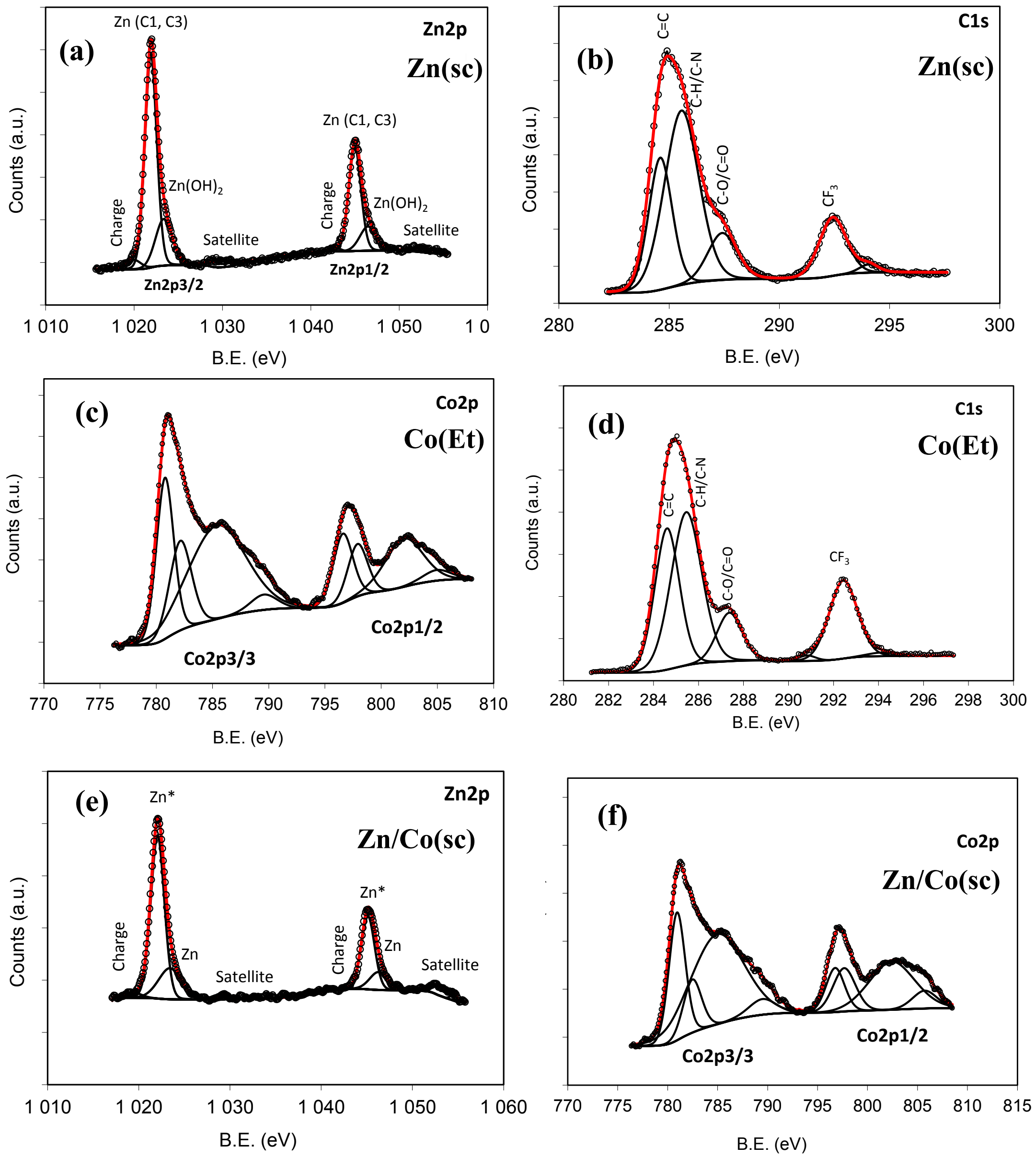

Figure S2. XPS regions for: (a) Zn2p and (b) C1s in Zn(sc) sample, (c) Co2p and (d) C1s in Co(Et) sample, and (e) Zn2p and (f) Co2p in Zn/Co(sc) sample.

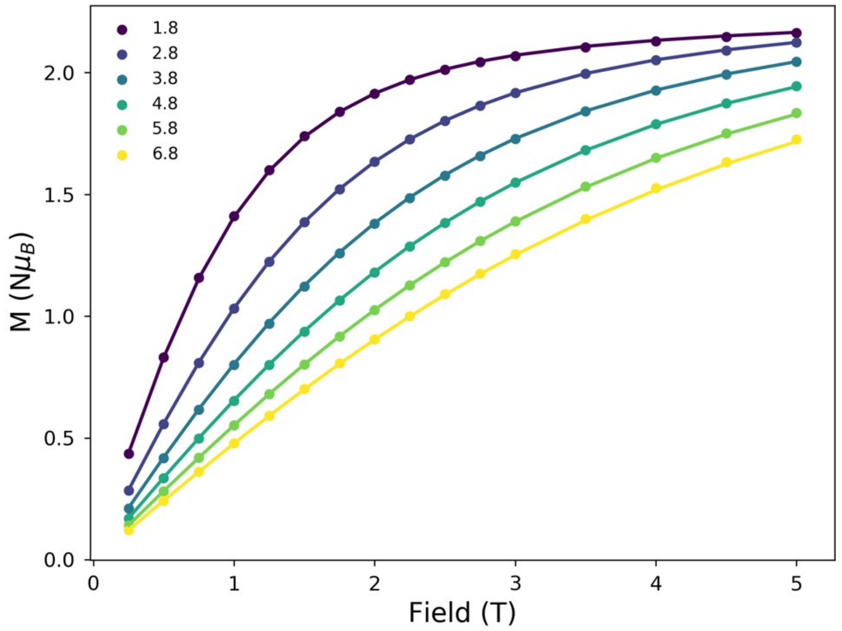

Figure S3. Molar magnetization *vs.* field for **Co(3)**.

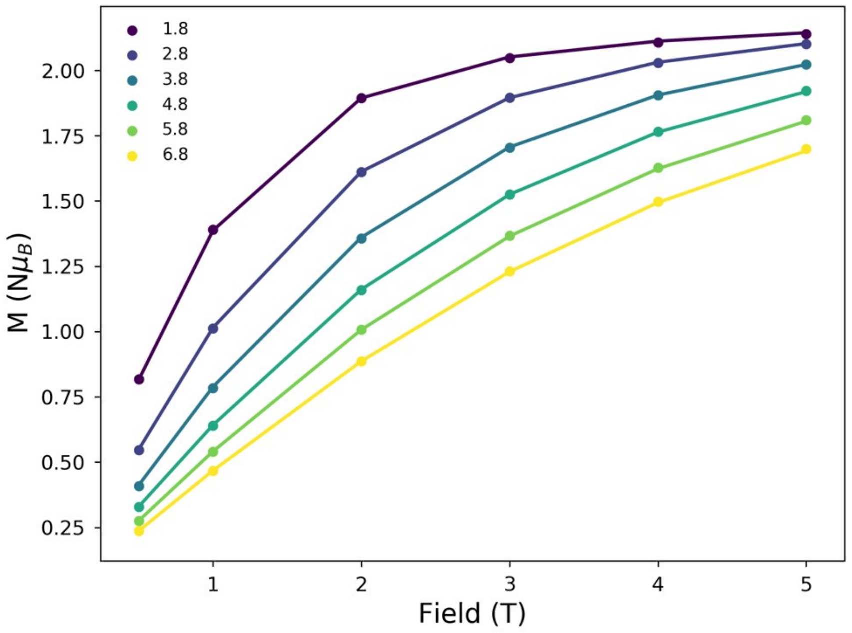

Figure S4. Molar magnetization *vs.* field for **Zn/Co(4)**.

(a)

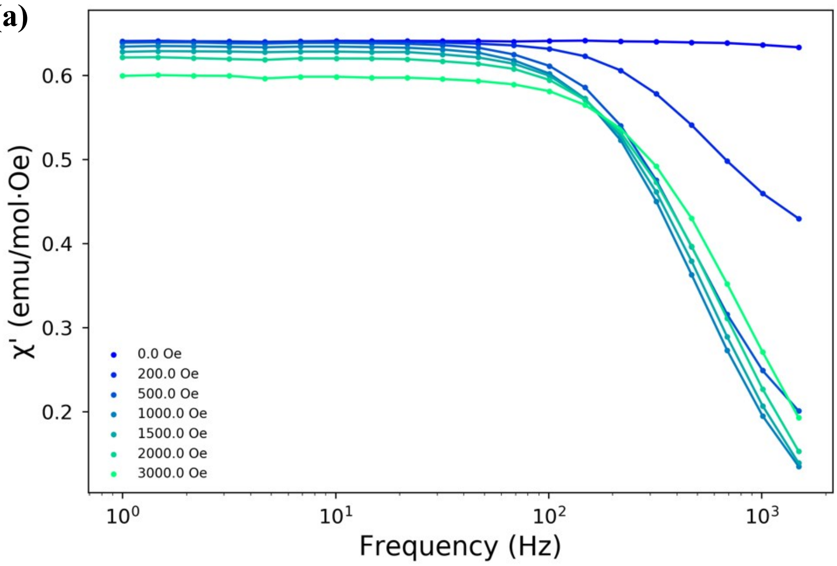

(b)

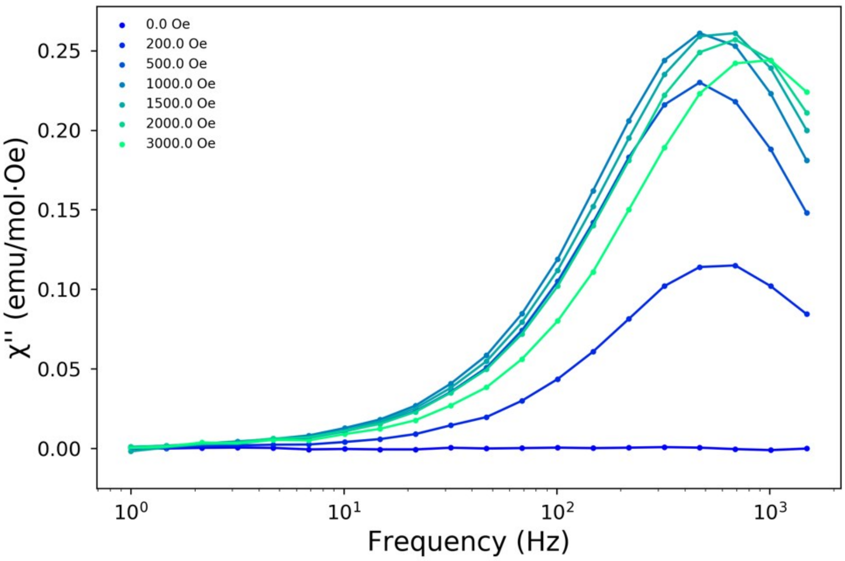

Figure S5. Frequency dependence of: (a) real and (b) imaginary parts of the magnetic susceptibility at 2.8 K and for different applied static dc fields for compound **Co(2).** The line is a guide for the eye.

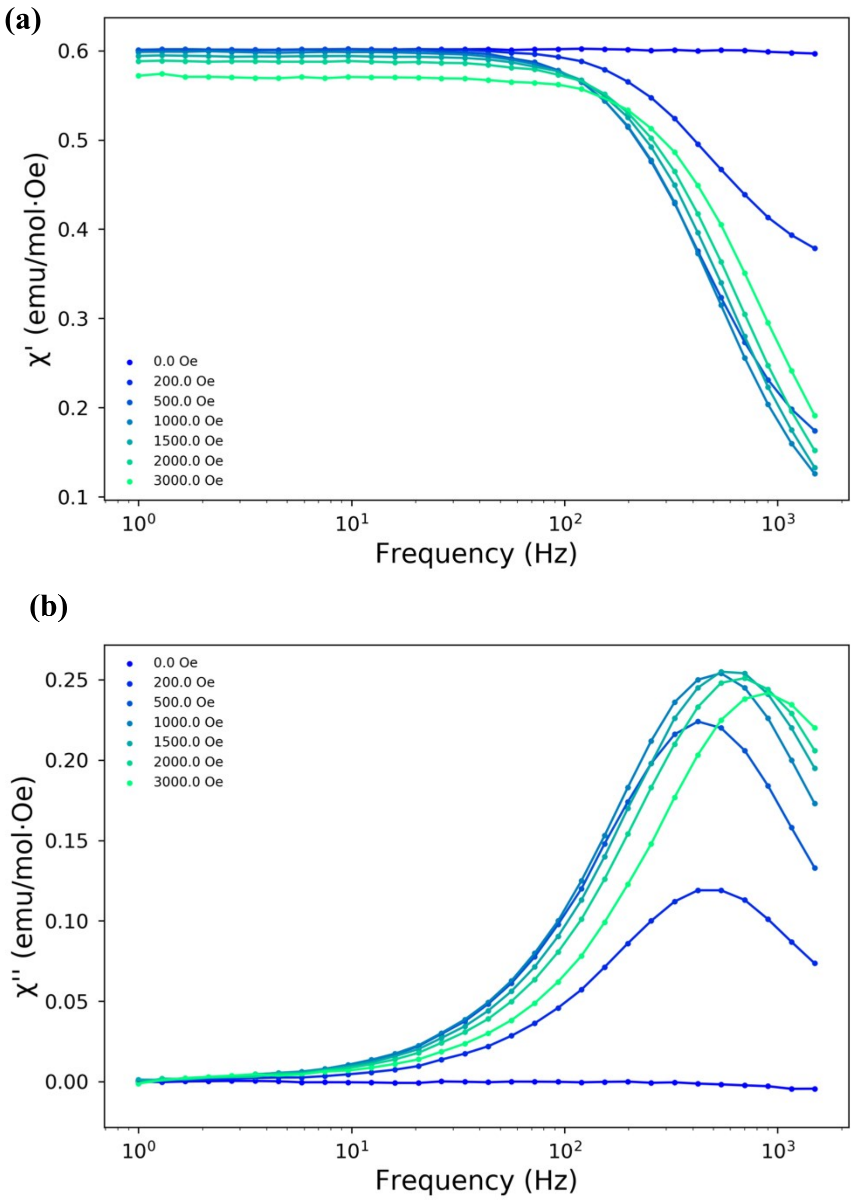

Figure S6. Frequency dependence of: (a) real and (b) imaginary parts of the magnetic susceptibility at 3.0 K and for different applied static dc fields for compound **Co(3)**. The line is a guide for the eye.

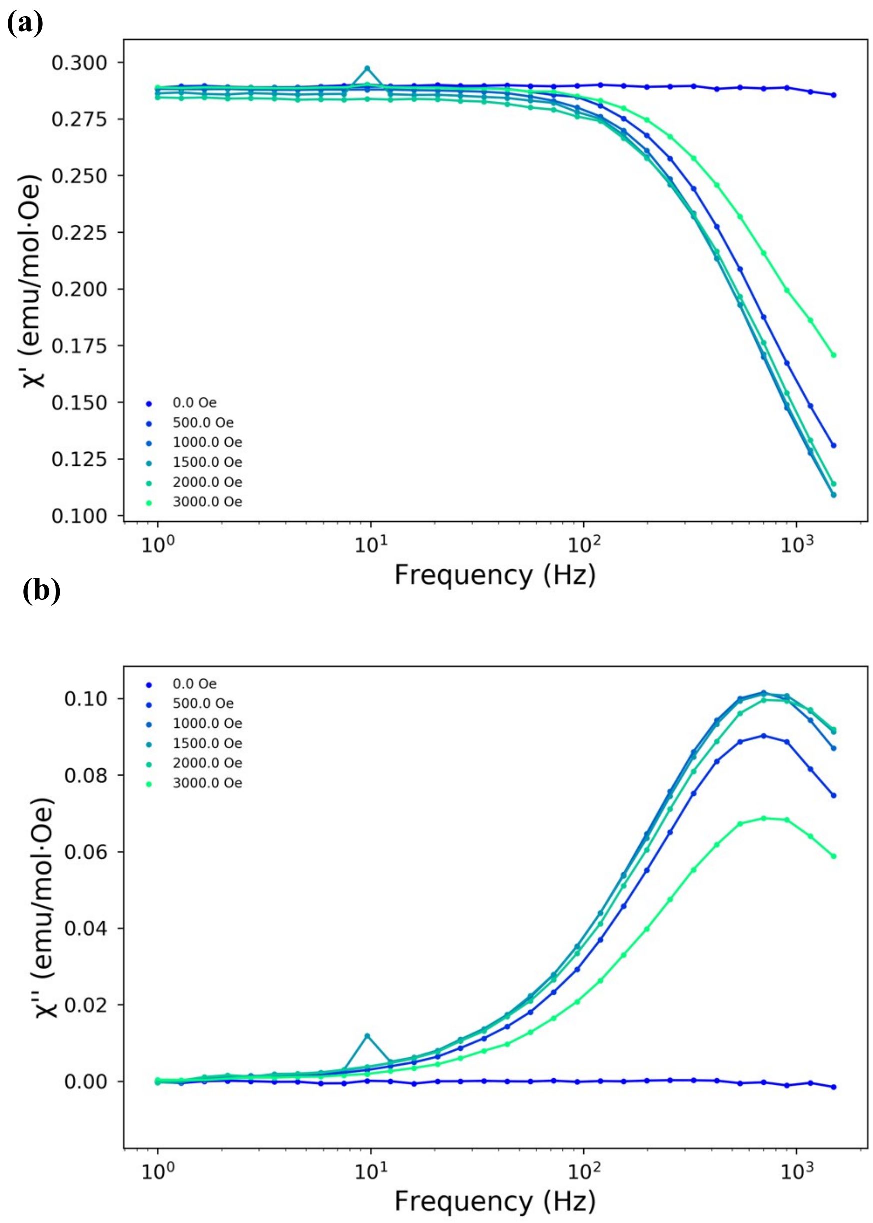

Figure S7. Frequency dependence of: (a) real and (b) imaginary parts of the magnetic susceptibility at 3.5 K and for different applied static dc fields for compound **Zn/Co(4)**. The line is a guide for the eye.

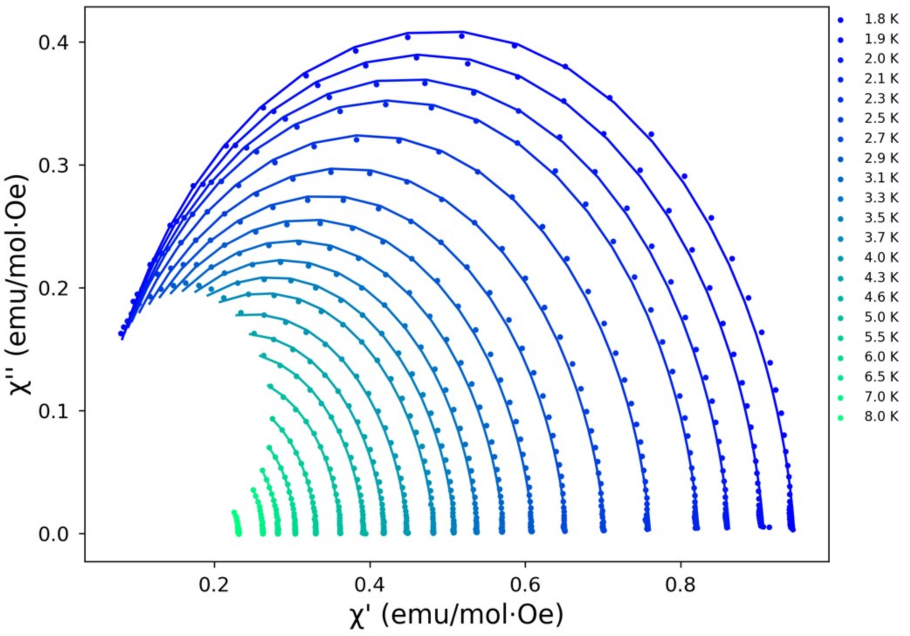

Figure S8. Cole-Cole plot at an applied static dc field of 1500 Oe and for different temperatures for compound **Co(2)**. The lines are the best fit to a Debye model using the CCfit package.

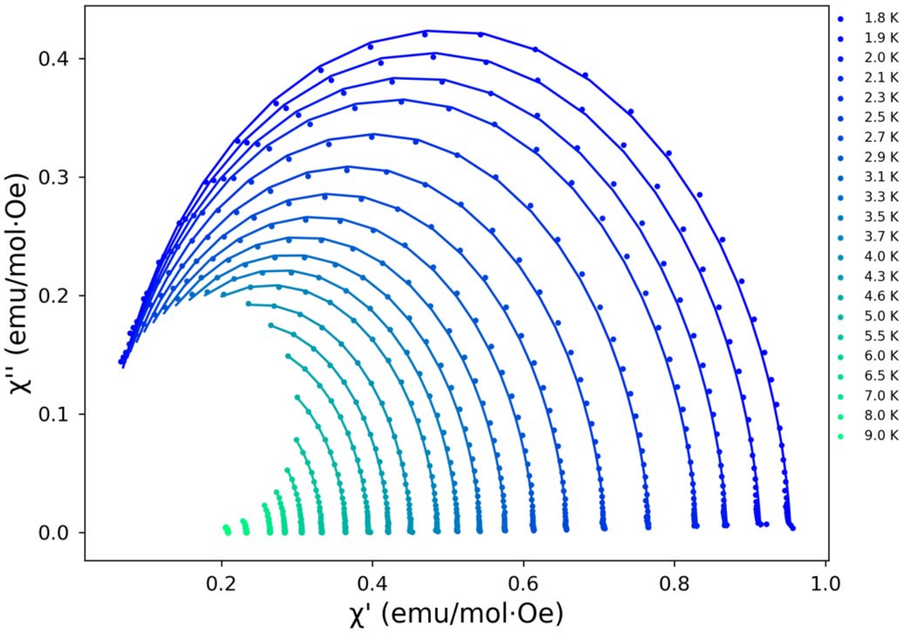

Figure S9. Cole-Cole plot at an applied static dc field of 1500 Oe and for different temperatures for compound **Co(3)**. The lines are the best fit to a Debye model using the CCfit package.

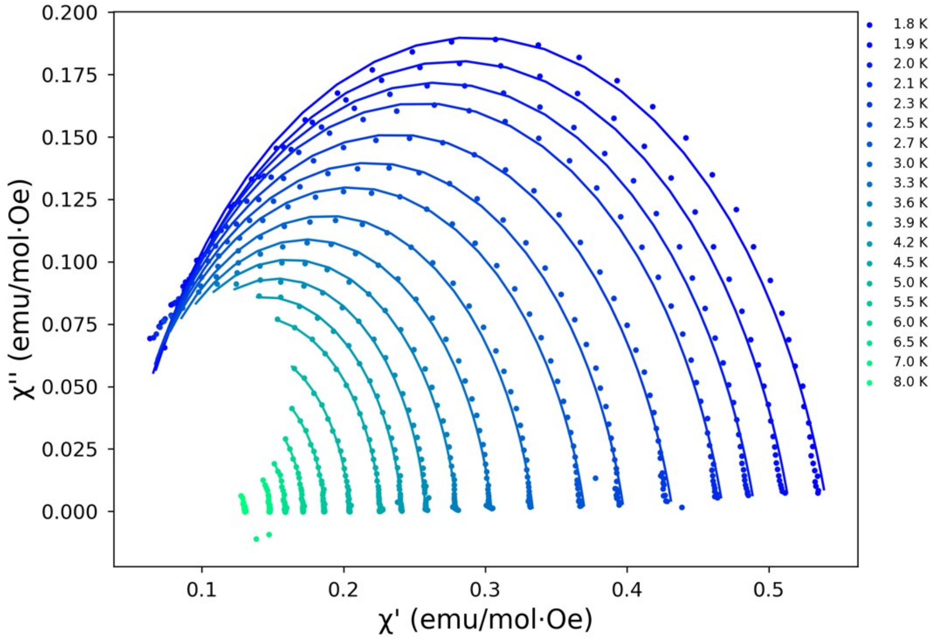

Figure S10. Cole-Cole plot at an applied static dc field of 1500 Oe and for different temperatures for compound **Zn/Co(4)**. The lines are the best fit to a Debye model using the CCfit package.

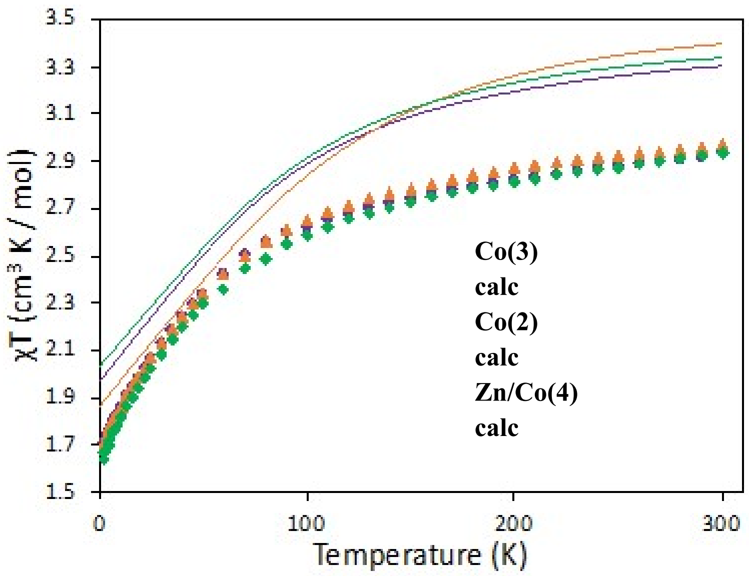

Figure S11. Comparison of the susceptibility and magnetization curves obtained from the CASPT2 calculations and experimental data.

REFERENCES

(1) Y.-C. Chiang, Y.-J. Chen, C.-Y. Wu, Effect of Relative Humidity on Adsorption Breakthrough of CO(2) on Activated Carbon Fibers, Materials, 10 (2017) 1296.

(2) D. Briggs, G. Beamson, Primary and secondary oxygen-induced C1s binding energy shifts in x-ray photoelectron spectroscopy of polymers, Analytical Chemistry, 64 (1992) 1729-1736.

(3) Z. Xing, Z. Ju, Y. Zhao, J. Wan, Y. Zhu, Y. Qiang, Y. Qian, One-pot hydrothermal synthesis of Nitrogen-doped graphene as high-performance anode materials for lithium ion batteries, Scientific Reports, 6 (2016) 26146.

(4) P. Han, Y. Yue, Z. Liu, W. Xu, L. Zhang, H. Xu, S. Dong, G. Cui, Graphene oxide nanosheets/multi-walled carbon nanotubes hybrid as an excellent electrocatalytic material towards VO2+/VO2+ redox couples for vanadium redox flow batteries, Energy & Environmental Science, 4 (2011) 4710-4717.

(5) Gharachorlou, M.D. Detwiler, A.V. Nartova, Y. Lei, J. Lu, J.W. Elam, W.N. Delgass, F.H. Ribeiro, D.Y. Zemlyanov, Palladium Nanoparticle Formation on TiO2(110) by Thermal Decomposition of Palladium(II) Hexafluoroacetylacetonate, ACS Applied Materials & Interfaces, 6 (2014) 14702-14711.

(6) R. O' Donoghue, D. Peeters, D. Rogalla, H.-W. Becker, J. Rechmann, S. Henke, M. Winter, A. Devi, Systematic molecular engineering of Zn-ketoiminates for application as precursors in atomic layer depositions of zinc oxide, Dalton Transactions, 45 (2016) 19012-19023.

(7) A. Fernández-Pérez, V. Rodríguez-Casado, T. Valdés-Solís, G. Marbán, Room temperature sintering of polar ZnO nanosheets: II-mechanism, Physical Chemistry Chemical Physics, 19 (2017) 16413-16425.

(8) G. Ahmed, M. Hanif, K. Mahmood, R. Yao, H. Ning, D. jiao, M. Wu, J. Khan, Z. Liu, Lattice defects of ZnO and hybrids with GO: Characterization, EPR and optoelectronic properties, AIP Advances, 8 (2018) 025218.

(9) B.R. Strohmeier, W.T. Evans, D.M. Schrall, Preparation and surface characterization of zincated aluminium memory-disc substrates, Journal of Materials Science, 28 (1993) 1563-1572.

(10) H. Darmstadt, C. Roy, S. Kaliaguine, Esca Characterization of Commercial Carbon-Blacks and of Carbon-Blacks from Vacuum Pyrolysis of Used Tires, Carbon, 32 (1994) 1399-1406.

(11) M.C. Biesinger, B.P. Payne, A.P. Grosvenor, L.W.M. Lau, A.R. Gerson, R.S.C. Smart, Resolving surface chemical states in XPS analysis of first row transition metals, oxides and hydroxides: Cr, Mn, Fe, Co and Ni, Applied Surface Science, 257 (2011) 2717-2730.
